# Wirelessly observed therapy compared to directly observed therapy to confirm and support tuberculosis treatment adherence: A randomized controlled trial

**DOI:** 10.1371/journal.pmed.1002891

**Published:** 2019-10-04

**Authors:** Sara H. Browne, Anya Umlauf, Amanda J. Tucker, Julie Low, Kathleen Moser, Jonathan Gonzalez Garcia, Charles A. Peloquin, Terrence Blaschke, Florin Vaida, Constance A. Benson

**Affiliations:** 1 University of California San Diego, La Jolla, California, United States of America; 2 Orange County Health Care Agency, Santa Ana, California, United States of America; 3 Health and Human Services Agency, San Diego, California, United States of America; 4 University of Florida, Gainesville, Florida, United States of America; 5 Stanford University, Stanford, California, United States of America; University of Cape Town, SOUTH AFRICA

## Abstract

**Background:**

Excellent adherence to tuberculosis (TB) treatment is critical to cure TB and avoid the emergence of resistance. Wirelessly observed therapy (WOT) is a novel patient self-management system consisting of an edible ingestion sensor (IS), external wearable patch, and paired mobile device that can detect and digitally record medication ingestions. Our study determined the accuracy of ingestion detection in clinical and home settings using WOT and subsequently compared, in a randomized control trial (RCT), confirmed daily adherence to medication in persons using WOT or directly observed therapy (DOT) during TB treatment.

**Methods and findings:**

We evaluated WOT in persons with active *Mycobacterium tuberculosis* complex disease using IS-enabled combination isoniazid 150 mg/rifampin 300 mg (IS-Rifamate). Seventy-seven participants with drug-susceptible TB in the continuation phase of treatment, prescribed daily isoniazid 300 mg and rifampin 600 mg, used IS-Rifamate. The primary endpoints of the trial were determination of the positive detection accuracy (PDA) of WOT, defined as the percentage of ingestions detected by WOT administered under direct observation, and subsequently the proportion of prescribed doses confirmed by WOT compared to DOT. Initially participants received DOT and WOT simultaneously for 2–3 weeks to allow calculation of WOT PDA, and the 95% confidence interval (CI) was estimated using the bootstrap method with 10,000 samples. Sixty-one participants subsequently participated in an RCT to compare the proportion of prescribed doses confirmed by WOT and DOT. Participants were randomized 2:1 to receive WOT or maximal in-person DOT. In the WOT arm, if ingestions were not remotely confirmed, the participant was contacted within 24 hours by text or cell phone to provide support. The number of doses confirmed was collected, and nonparametric methods were used for group and individual comparisons to estimate the proportions of confirmed doses in each randomized arm with 95% CIs. Sensitivity analyses, not prespecified in the trial registration, were also performed, removing all nonworking (weekend and public holiday) and held-dose days. Participants, recruited from San Diego (SD) and Orange County (OC) Divisions of TB Control and Refugee Health, were 43.1 (range 18–80) years old, 57% male, 42% Asian, and 39% white with 49% Hispanic ethnicity. The PDA of WOT was 99.3% (CI 98.1; 100). Intent-to-treat (ITT) analysis within the RCT showed WOT confirmed 93% versus 63% DOT (*p* < 0.001) of daily doses prescribed. Secondary analysis removing all nonworking days (weekends and public holidays) and held doses from each arm showed WOT confirmed 95.6% versus 92.7% (*p* = 0.31); WOT was non-inferior to DOT (difference 2.8% CI [−1.8%, 9.1%]). One hundred percent of participants preferred using WOT. WOT associated adverse events were <10%, consisting of minor skin rash and pruritus associated with the patch. WOT provided longitudinal digital reporting in near real time, supporting patient self-management and allowing rapid remote identification of those who needed more support to maintain adherence. This study was conducted during the continuation phase of TB treatment, limiting its generalizability to the entire TB treatment course.

**Conclusions:**

In terms of accuracy, WOT was equivalent to DOT. WOT was superior to DOT in supporting confirmed daily adherence to TB medications during the continuation phase of TB treatment and was overwhelmingly preferred by participants. WOT should be tested in high-burden TB settings, where it may substantially support low- and middle-income country (LMIC) TB programs.

**Trial registration:**

ClinicalTrials.gov NCT01960257.

## Author summary

**Why was this study done?:**

- TB is the infectious disease that kills the most people annually and is spread by droplets released into the air from persons with TB in their lungs.
- Multiple antibiotics taken over many months are necessary to cure this infection, and if doses are missed or full treatment is not completed, infected people can continue spreading the disease, and antibiotic resistance may occur.
- New methods to confirm that all the correct medication doses are taken and support patients over the long treatment course are crucial to saving lives and combatting disease spread.

**What did the researchers do and find?:**

- We tested a new FDA-approved technology that uses a sensor made of minerals that is swallowed with TB medication and subsequently records the ingestion on a cellphone.
- We tested the sensor system accuracy to identify ingestions and we also randomly assigned persons with TB to receive the new system or the standard of care currently available, directly observed therapy, for supporting and confirming medication taking during TB treatment for periods up to 29 weeks.
- We found the system was highly accurate and that persons using this system were confirmed as taking 93% of their daily prescribed doses as opposed to 63% using directly observed therapy. In this study, the difference observed was mainly driven by the ability of the system to confirm doses 7 days a week compared to only on working days for directly observed therapy.

**What do these findings mean?:**

- These findings indicate that novel sensor-based systems that use cell phones to remotely record digital data are as accurate as actually watching a person swallow their medications and can be used to confirm and support TB treatment 7 days a week, compared to 5 days a week for the current standard of care available. However, this technology still needs to be tested over the full course of TB treatment.
- WOT should be tested in high-burden TB settings, where it may substantially support LMIC TB programs.

## Introduction

*Mycobacterium tuberculosis* complex infects a quarter of the world’s population. Of these, active tuberculosis (TB) is present in 10 million people, causing death in 1.4 million of those in 2017 [1]. Of all infections, TB kills the highest number of people worldwide and is the leading cause of death among persons living with HIV infection globally [1].

Major advances have taken place in TB treatment in the last decade. These include the detection of infection by interferon gamma release assay (IGRA) with the implementation of QuantiFERON-TB-Gold testing, and rapid diagnostics using cartridge-based nucleic acid amplification, allowing point-of-care identification of TB DNA and *rpoB* mutations with the implementation of GeneXpert [2]. Novel oral drugs have been developed for multidrug-resistant TB (MDR-TB), such as delamanid and bedaquiline; other antibiotics such as clofazimine and linezolid have been repurposed, enabling the first-ever entirely oral regimen for MDR-TB [3]. New classes of antibiotics have been added to standard regimens for sensitive TB [4]. However, innovation in TB treatment adherence support and reporting has lagged, threatening the efficacy of strides made in TB genomics and pharmaceutics.

Poor adherence to TB treatment is associated with delays in sputum conversion and thus continued transmission, lower treatment completion or default with disease relapse, and the emergence of MDR-TB [5]. Multiple lines of evidence indicate the need for improved treatment adherence support. Missed doses were a significant risk factor amongst defaulters in new smear-positive patients treated under short-course directly observed therapy (DOTS) in India [6]. Relapse rates are high within several TB programs in high-burden countries such as India (15%–18%) [7, 8] and South Africa (17%) [9] and in MDR-TB programs in Eastern Europe (44%) [10]. In South Africa, den Boon and colleagues reported that previously treated individuals represented more than half of the patients with smear-positive TB, indicating that previously treated cases contribute to the ongoing transmission of TB within such communities [11]. In such high-burden areas, it can be difficult to distinguish between relapse and reinfection. Data from the same area of South Africa support the contention that over 50% of cases are relapses; specifically, in evaluating 276 recurrent TB cases for which DNA fingerprints were available for both the index and recurrent case, 52% were confirmed as relapse cases [9]. Recurrence risks that are associated with poor adherence or early discontinuation have been difficult to quantitate. Irregular medication adherence was associated with relapse, and the degree of nonadherence was strongly associated with increasing risk of recurrence 18 months after completing treatment in programmatic care in India [7]. Poor adherence has also been identified as a major factor in the emergence of MDR-TB. Cadosch and colleagues estimate the risk of generating de novo MDR-TB is highest between 40%–80% adherence [12].

The impact of TB medication dosing frequency and the importance of daily dosing was confirmed in a recent meta-analysis of all key fluoroquinolone treatment shortening trials, TB ReFLECT [12]. Not only did patients taking four-drug therapy with less than 90% adherence have a 5.6 increased risk of TB recurrence [13], but Kaplan–Meier estimates showed study participants who fully adhered to a dosing regimen of 6 out of 7 days per week (6/7) had a higher probability of unfavorable outcome than those who adhered to and completed a dosing regimen of 7 of 7 days per week (7/7) (HR, 2.7; 95% confidence interval [CI], 1.1–6.7, after adjustment for treatment duration and country). These data substantiate earlier evidence that daily dosing of TB medication is more effective in both HIV-uninfected [14, 15] and HIV-coinfected individuals [16]. Clinical findings on the impact of daily dosing are confirmed by nonclinical data from the hollow fiber system [17]. Drusano and colleagues [17] evaluated the effect of 5 of 7 days of therapy (5/7-day regimen) using combined rifampicin and moxifloxacin. These agents have discordant half-lives (1.9 versus 6.5 hours when employed in combination), and moxifloxacin induces error-prone replication in *Mycobacterium tuberculosis*. They demonstrated that 5 of 7 days of therapy (5/7-day regimen) allows the emergence of resistance to moxifloxacin, which was not seen with 7/7-day therapy. Drusano and colleagues express concern for “drug holidays” associated with 5/7 days of therapy.

However, directly observed therapy (DOT), currently the method of the highest standard recommended to ensure treatment adherence and reporting, is clinically available 5/7 days of therapy at best. In DOT, a healthcare worker observes the swallowing of the medication and provides written verification of treatment adherence and completion. DOT provided by a healthcare worker in the community is superior to in-clinic or family DOT and self-administration therapy (SAT) in achieving treatment success [5]. Digital health interventions such as text messaging and electronic pill-boxes have shown improvements in treatment success in comparison to their absence [5] but have not been compared to in-person DOT. One study of the telemedicine video observed therapy (VOT) did find the number of appointments kept for VOT were comparable to the numbers of appointments kept for in-person DOT and could allow health workers to observe a similar number of patients to in-clinic DOT [18].

While DOT remains the reference standard, it is resource-intensive, difficult to achieve—particularly over geographical distances—time-consuming, and represents the largest single cost of TB treatment [19]. DOT has been described as intrusive and disempowering for patients [19]. All the relapse rates quoted above in high-burden areas had DOT in some form implemented. Where DOT is offered in high-burden global settings, it is largely limited to in-clinic DOT, which is still burdensome and costly in environments with a limited number of healthcare workers, and to patients who may have to miss work, travel, or leave children or elder relatives [20, 21]. In the United States, maximal DOT administration is carried out 5 days each week, and data indicate that even in this low-burden setting, only 18% of patients complete treatment with DOTS within 6 months [22]. DOT has also been associated with adaptations such as once-weekly [23] and bi- and tri-weekly medication dosing [24, 25]. These adaptations result in variation in the amount of drug taken over a weekly period (in the case of rifampin in the US, these are 1,200 mg for bi-weekly and 1,800 mg for tri-weekly, in comparison to 4,200 mg for daily dosing). Clinically, currently implemented DOT is a considerable departure from the original data supporting the short course of pulmonary TB treatment, in which patients received “chemotherapy in hospital to assure regular administration” 7 days a week [26]. And although medication is prescribed daily when DOT can be implemented 5 days a week, because weekend doses cannot be confirmed, they are not documented or counted by public health personnel in their treatment logs. Essentially, confirmation of actual drug ingestion on a daily basis has not been possible until recently.

A novel sensor platform to monitor medication ingestion (Proteus Digital Health, Redwood City, CA, USA) presents the opportunity to capture daily medication ingestion digitally and analyze and support medication adherence in near real time. This system, termed wirelessly observed therapy (WOT) in the context of TB treatment adherence support, allows date- and time-stamping of actual medication ingestion. The system consists of an ingestion sensor (IS)—approximately 1 mm^3^ (1 × 1 × 0.45 mm)—coated with very thin layers of commonly ingested excipients (that is, minerals and metals) (S1 Fig), a small adhesive-backed detector patch worn on the torso, and a paired mobile device. When ingested with a medication, the sensor readily separates from the carrier, is energized, and communicates with the detector patch [27]. The detector patch interprets the information as unique to the ingested sensor. The detector patch can also record physiological metrics. Data from the patch are transmitted wirelessly, via Bluetooth technology, to a paired device such as a mobile phone, tablet, or personal computer. Subsequently, all of the data on the paired device are uploaded to a secured, centralized data storage location [27] (S2 Fig). These data are available in near real time to the patient user on their mobile device and, with patient permissions, to healthcare personnel and other significant persons, who can access these data from a secure web portal [28–31] (S2 Fig).

The significance of this technology for TB treatment support was recognized during early testing of the system prior to its full development [32]. This system currently serves as a patient self-management system, to increase patient empowerment and control, and to allow support from significant others and healthcare workers with permissions across multiple disease states managed with oral medication (S2 Fig) [29, 30, 33–35]. Now that the IS platform is fully developed, it is possible for TB treatment programs, with patient permissions, to follow large cohorts of patients using the secure web-based dashboard and provide support in near real time such that intervention, if needed, can be provided in a highly targeted manner. We tested WOT in patients with active tuberculosis in collaboration with two public health treatment programs in southern California, USA. We developed an IS-enabled combination isoniazid 150 mg/rifampin 300 mg (IS-Rifamate) and performed a study to validate bioequivalence in actively infected TB patients [34]. Here, we report the results of the first trial of WOT in patients with active TB disease. Initially, the accuracy of WOT using IS-Rifamate in clinical and home settings was established using simultaneous DOT and WOT. Subsequently, in a randomized study, the ability of WOT to confirm and support continuous adherence to TB medication was compared to DOT.

## Methods

The study was open-label and prospective, consisting of evaluating the accuracy of WOT (Stage 1) and randomized (Stage 2) comparison of the ability of WOT and DOT to confirm and support adherence to TB continuation phase treatment. The primary endpoints of the trial were determination of i) the positive detection accuracy (PDA) of WOT, defined as the percentage of IS-Rifamate ingestions detected by WOT when administered under direct observation, and subsequently ii) the proportion of prescribed doses confirmed by WOT in comparison to DOT. In Stage 1, all subjects received two tablets of IS-Rifamate [36] witnessed by in-person DOT to determine the detection accuracy of WOT in the clinical and home environment. In Stage 2, subjects were randomized to intervention with WOT IS-Rifamate or control within the public healthcare system standard of care (SOC) 5-days-a-week DOT. Randomization was centrally generated via an algorithm residing within the study database. Randomization into arms followed a 2:1 allocation, stratifying by study site (San Diego [SD] County and Orange County [OC]). Specifically, a block of size 3 was used; within each block, 2 participants were randomized to WOT, and 1 participant was randomized to DOT. Subjects were recruited from SD and OC Divisions of TB Control and Refugee Health, which were both study sites, in addition to the Anti-Viral Research Center (AVRC) at University of California SD (UCSD). The protocol (“AHF TB 001 Wirelessly Observed Therapy in Comparison to Directly Observed Therapy for the Treatment of Tuberculosis”; ClinicalTrials.gov NCT01960257) was approved by the UCSD Internal Review Board (#130841), the County of Orange Health Care Agency Human Subjects Review Committee (# 2014–06), and the County of SD Health and Human Services Agency (#121274X) and was conducted in accordance with the principles of good clinical practice. All information regarding procedures, risks, and data privacy was made known to the participants before obtaining signed consent. This study is reported as per the Consolidated Standards of Reporting Trials (CONSORT) guideline (see S1 CONSORT Checklist).

### Research compliance

Study participants were adult TB patients in the continuation phase of treatment who were smear-negative and taking isoniazid plus rifampin, with no evidence of drug-resistant TB, and who had complete blood count (CBC) and comprehensive panel (CMP) values within defined parameters (absolute neutrophil count [ANC] ≥ 1,000/mm^3^; hemoglobin ≥ 9.0 g/dL; platelet count ≥ 75,000/mm^3^; AST [SGOT], ALT [SGPT], and alkaline phosphatase ≤ 3 × ULN; total bilirubin ≤ 1.5 × ULN and direct bilirubin). Participants had to be able to understand written or verbal information regarding WOT, be able to use a mobile device, and be willing to wear a patch. Criteria for exclusion were pregnancy and known hypersensitivity to skin adhesives.

**Fig 1:**
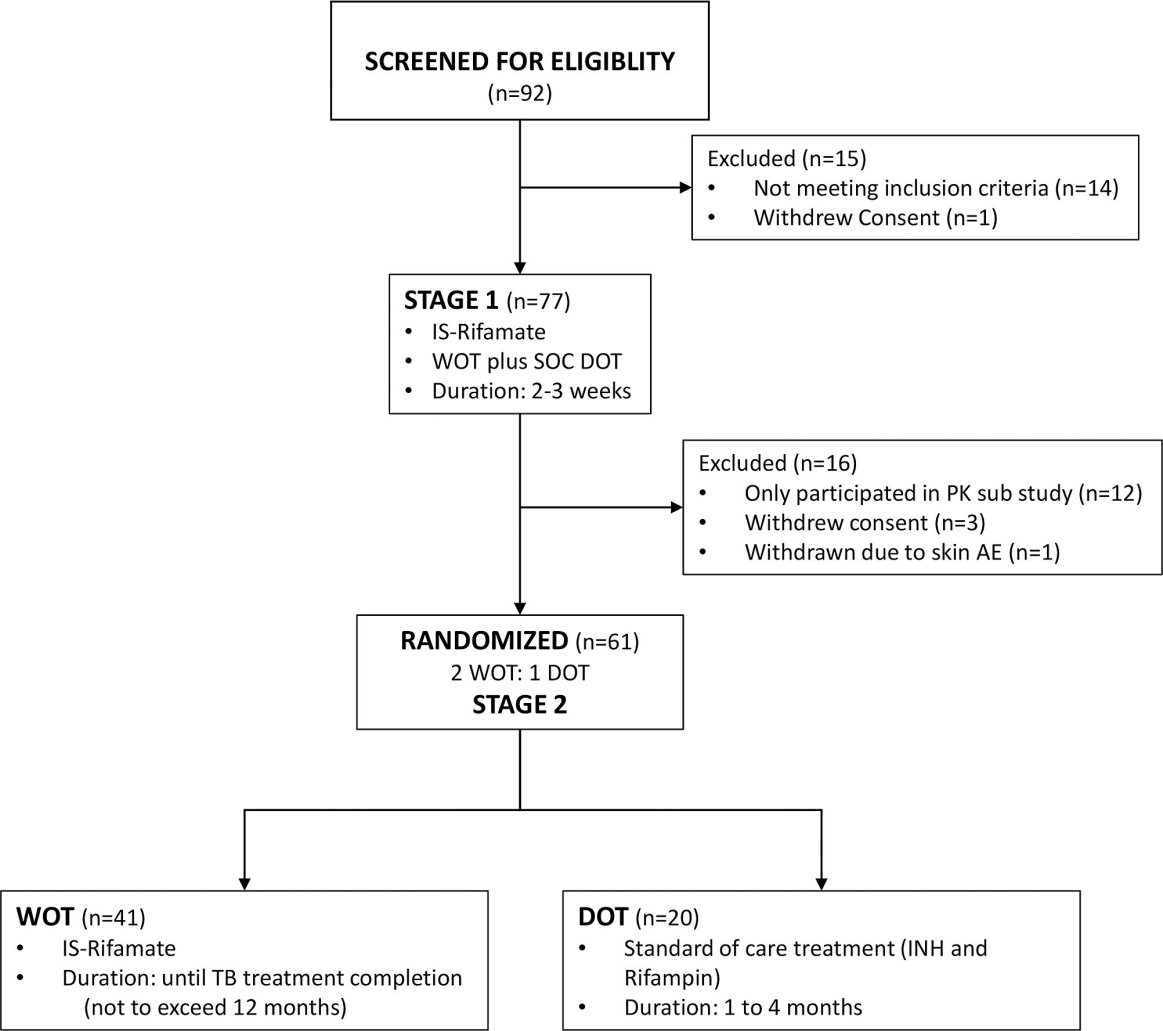
Study flow sheet. AE, adverse event; DOT, directly observed therapy; INH, isoniazid; IS, ingestion sensor; IS-Rifamate, IS-enabled combination isoniazid 150 mg/rifampin 300 mg; PK, pharmacokinetic; SOC, standard of care; TB, tuberculosis; WOT, wirelessly observed therapy.

In Stage 1, data were gathered from 77 participants enrolled between October 2013 and January 2017; this period included the performance of a bioequivalence study in which 12 persons were coenrolled (see Fig 1) (34). Sixty-one participants were subsequently randomized 2:1 to WOT with IS-Rifamate alone or to county SOC with healthcare-worker–delivered 5-day-a-week DOT using standard isoniazid plus rifampin. Stage 1 was 2–3 weeks in duration and included training on WOT use and patch changes. In Stage 2, participants randomized into the WOT arm changed the monitor patch approximately weekly by themselves. The participants were able to view their own medication ingestion log on the mobile device. Study staff and county health workers checked the ingestion log remotely on the Proteus Digital Health website on business days to confirm dosing. If ingestion of IS-Rifamate was not confirmed on any business day, the patient was contacted by text that day, followed by a phone call within 24 hours. In Stage 2, participants using WOT were seen 2 weeks after randomization and then monthly. Initially, participants in both arms were to be followed for 16 weeks. However, because participants using WOT did not want to go back onto DOT, OC and SD County requested that they be allowed to continue WOT until the end of the participants’ treatment course; thus, the length of observed treatment varied. All participants were followed medically by their treating physicians and assigned county healthcare workers.

The study flow diagram is shown in Fig 1.

### Statistical analysis

Sample size calculations were performed during the planning phase of the study for the comparison of WOT and DOT arms’ abilities to confirm doses. The primary endpoint for these analyses was estimated for each participant by a single value for the percent of confirmed doses for the duration of their stay in the study. Thus, for each arm, an average proportion of confirmed doses can be calculated, and, assuming a normal distribution for this endpoint, it is appropriate to evaluate the overall absolute difference between arms using parametric methods. Sample size calculations were based on a two-sided, two-sample *t* test to compare the differences in confirmed individual-level dose percentages by treatment arm using different mean/standard deviation scenarios. Assuming a two-sided alpha of 0.05, a percentage of confirmed doses in the DOT arm of 90%, and a common standard deviation of 6%, we calculated needing 75 participants (50 WOT arm and 25 DOT arm) to detect a 5% difference (that is, 95% adherence percentage in WOT arm) with 92% power. After the data were collected, the calculated percentage of confirmed doses had a negatively skewed distribution, and its average approached the ceiling of 100% for the WOT arm; therefore, a decision was made to use nonparametric methods instead of the *t* test to compare the differences in confirmed individual-level dose percentage. The nonparametric analyses we used in testing our hypotheses are described in detail below.

To evaluate WOT accuracy in Stage 1, the primary endpoint was the PDA of WOT in the clinical and home setting, where PDA was defined as the number of sensor-detected IS-Rifamate doses divided by the total number of simultaneously directly observed doses. In this as-treated analysis, a complete dose was defined as 1 or more tablets detected by WOT. Exclusions were days when DOT was not administered or the WOT system was not used properly (that is, the patch not worn or not connected). The 95% CI for PDA was estimated using a bootstrap method with 10,000 samples. Each bootstrap sample involved two stages. First, *n* = 77 participants were sampled with replacement from among the study participants. Then, the data were resampled with replacement from the observed response vector of each participant separately. This procedure preserves the random variation between participants, and the correlation within participant [37]. The 95% CI was chosen as the middle 95% of the bootstrap distribution of the PDA proportions. Due to the fact that PDA is close to 100%, the initially planned parametric methods relying on normal approximations (e.g., GEE or generalized linear mixed-effects models) are not applicable.

For Stage 2, participants’ demographic characteristics were compared between arms using Wilcoxon rank–sum test for numeric variables and Fisher’s exact test for categorical variables. The primary endpoint of Stage 2 compared the ability of WOT and DOT to confirm and support INH/rifampin dosing over the time period of the study. The primary outcome was, for each participant and each day, the binary response of whether the doses were confirmed either wirelessly (WOT arm) or directly by a health worker (DOT arm). The WOT dose was defined as 1 or more tablets detected; sensitivity analyses were performed, defining a confirmed dose as 2 tablets detected. The confirmed doses were compared between arms in an intent-to-treat (ITT) analysis, taking into account the correlation of these binary outcomes within an individual. In this conservative approach, all follow-up days of observation were considered (4,022 in WOT and 1,904 in DOT) with no exclusions. Thus, improper use of the WOT system (that is, patch not worn or not connected) were included as nonconfirmed dose for WOT. Similarly, days when a healthcare worker was not available, including weekends, were counted as a nonobserved dose for DOT. The proportions of confirmed doses were estimated for each arm as the total number of confirmed doses over the total number of prescribed doses. The 95% CI and the associated *p*-value for the difference in confirmed doses were estimated using a bootstrap method with 10,000 samples. For each bootstrap sample, the *n* = 61 participants were sampled with replacement from among the Stage 2 participants. Then, the data were resampled with replacement from the observed response vector of each participant separately. This procedure preserves the random variation between participants and the correlation within participant [37]. The odds ratio (OR) and its 95% CI were estimated from these data. The 95% CI for each quantity of interest was chosen as the middle 95% of the bootstrap distribution of that estimator. Similarly, the *p*-value was computed as the two-sided tail probability of the estimated value relative to its bootstrap distribution. Due to the fact that for certain analyses, individual-level proportions are close to 100%, the initially planned parametric methods relying on normal approximations (e.g., GEE or generalized linear mixed-effects models) are not fully reliable. Sensitivity analyses using mixed-effects logistic regression were performed and gave consistent results (not included). Secondary, as-treated analyses were performed, excluding from calculations days when medication doses were held. In additional sensitivity analyses, the participant-level observed proportions were compared between arms using the Wilcoxon rank–sum test. Fisher’s exact test was used to compare the proportion of patients in each group whose individual confirmed daily dosing was 90% or higher. The 95% CIs for the differences of proportions and ORs comparing the two arms were based on the same bootstrap with 10,000 samples described above. In response to reviewers, additional secondary analyses were performed: i) a comparison of confirmed doses that removed all traditional nonworking days (that is, weekends and public holidays) from the DOT arm and doses held for medical reasons from both arms and ii) an analysis of confirmed adherence over time in both treatment arms to evaluate evidence of potential treatment fatigue associated with WOT over time. These time-trend analyses used a mixed-effects logistic regression, with subject-level random intercept, a linear time trend, treatment, and time-by-treatment interaction.

## Results

### Demographics

The demographic characteristics of the cohorts, shown in Table 1, are as follows: participants were on average 43.1 years old (range: 18–80 years) and 57% were males, mostly white (39%) or Asian (42%), with approximately half of them reporting Hispanic ethnicity (49%). Twenty-five percent had no high school (HS) diploma (or general education development [GED]), 35% had HS/GED, 26% had some technical or college education, 10% had a bachelor’s degree, and 4% further advanced education. Fifty-eight percent reported being unemployed or disabled, and 43% of those who responded to an inquiry about income reported a monthly household income well below the federal poverty level. English was the primary language for 28.6%. The participants randomized in Stage 2 to WOT or SOC DOT did not differ in demographic characteristics.

**Table 1:** Baseline demographic characteristics of participants enrolled in Stage 1 (*N* = 77) and Stage 2 (*N* = 61).

		Stage 1	Stage 2		
Demographic Characteristic		All Participants (*N* = 77)	WOT (*N* = 41)	DOT (*N* = 20)	*p*-Value^a^
Age, years	Mean (SD)	43 (17)	41 (16)	45 (17)	0.26
Male, *N* (%)		44 (57%)	21 (51%)	12 (60%)	0.25
Race, *N* (%)	African-American	1 (1%)	0 (0%)	0 (0%)	0.96
	Asian	32 (42%)	17 (41%)	8 (40%)	
	Pacific Islander	1 (1%)	1 (2%)	0 (0%)	
	White	30 (39%)	14 (34%)	8 (40%)	
	Unknown^b^	13 (17%)	9 (22%)	4 (20%)	
Ethnicity, *N* (%)	Hispanic/Latino	38 (49%)	21 (51%)	11 (55%)	1
	Not Hispanic/Latino	36 (47%)	18 (44%)	8 (40%)	
	Unknown	3 (4%)	2 (5%)	1 (5%)	
Education, *N* (%)	Less than HS	19 (25%)	10 (24%)	4 (20%)	0.96
	HS/GED	27 (35%)	14 (35%)	9 (45%)	
	Some college/technical	20 (26%)	10 (24%)	5 (25%)	
	Bachelor’s	8 (10%)	5 (12%)	2 (10%)	
	Advanced	3 (4%)	2 (5%)	0 (0%)	
Employment, *N* (%)	Full-time	21 (27%)	12 (29%)	6 (30%)	0.69
	Part-time	8 (10%)	5 (12%)	2 (10%)	
	Unemployed	32 (42%)	18 (44%)	7 (35%)	
	Retired	4 (5%)	0 (0%)	1 (5%)	
	Unable to work (disabled)	12 (16%)	6 (15%)	4 (20%)	
Average monthly household income^c^, *N* (%)	≤$100	20 (26%)	10 (24%)	7 (35%)	0.42
	$101–$500	4 (5%)	0 (0%)	2 (10%)	
	$501–$1,000	9 (12%)	2 (5%)	2 (10%)	
	$1,001–$2,000	13 (17%)	8 (20%)	3 (15%)	
	$2,001–$3,000	7 (9%)	6 (15%)	1 (5%)	
	$3,001–$4,000	1 (1%)	1 (2%)	0 (0%)	
	$4,001–$5,000	0 (0%)	0 (0%)	0 (0%)	
	≥$5,000	2 (3%)	2 (5%)	0 (0%)	
	Declined to answer	21 (27%)	12 (29%)	5 (25%)	

^a^Comparisons between WOT and DOT arms in Stage 2; Wilcoxon rank–sum test was used to compare age, Fisher’s exact test was used for other variables.

^b^Unknown includes 3 categories: “declined to answer,” “does not know,” and “not available.” The following categories are not shown because all had *N* = 0: American Indian, Native Alaskan, and Native Hawaiian.

^c^All employment groups are included.

**Abbreviations:** DOT, directly observed therapy; GED, general education development; HS, high school; SD, standard deviation; WOT, wirelessly observed therapy.

### Accuracy of WOT in the clinical and home setting

A total of 1,073 person-days of data were collected from the 77 participants enrolled in Stage 1. DOT and WOT were administered simultaneously on 685 person-days. The estimated PDA was 0.993 (680/685), and the 95% CI for the proportion of WOT doses detected when witnessed by DOT was 0.981 and 1.000. DOT was absent on 286 weekend days, 30 national or state holidays, and 64 days when DOT was missing for unspecified reasons, and WOT was absent on 8 days when the IS-Rifamate dose was not recorded because of incorrect use of the system (that is, monitor patch not worn or not connected to iPad [6], DOT done in error [2]).

### Dosing confirmation and maintenance of adherence of WOT in comparison to DOT within the randomized controlled study

The study duration for each participant in Stage 2, defined as the total number of days under treatment, varied because of comorbidities and cases in which the public health department requested to continue participants on WOT until the end of their treatment (these patients declined to go back on DOT). Table 2 shows treatment duration by arm. The median duration of participation in Stage 2 was 99 days, ranging from 12 to 206, days with no statistical difference between WOT and DOT arms (median 93 versus 101, *p* = 0.85).

**Table 2:** Treatment duration (days) in Stage 2 for the entire cohort and by treatment arm.

	All	WOT	DOT	*p*-Value^a^
Total number of participants, *N*	61	41	20	
Total number of observations	5,926	4,022	1,904	
Days	99 (61, 116)	93 (58, 127)	101 (88, 113)	0.85
Median (IQR) range	12–206	12–206	25–155	

^a^Comparisons using Wilcoxon rank–sum test.

**Abbreviations:** DOT, directly observed therapy; IQR, interquartile range; WOT, wirelessly observed therapy.

A total of 5,926 observation days were used, with 1,904 in the DOT arm and 4,022 in the WOT arm. Table 3 shows results of the ITT analysis for the primary comparisons of treatment effectiveness to confirm the daily dose between WOT and DOT arms. On the group level, 92.9% (3,738 out of 4,022) of prescribed doses were confirmed in the WOT treatment, significantly different (*p* < 0.001) from the 63.1% (1,202 out of 1,904) of prescribed doses observed in the DOT arm. Participant-level sensitivity analyses showed similar results (*p* < 0.001).

**Table 3:** Proportion of confirmed doses, WOT versus DOT. ITT analysis. Confirmed dose defined as 1 or more tablets detected or witnessed.

	WOT (*N* = 41)	DOT (*N* = 20)	Difference (95% CI)	OR (95% CI)	*p*-Value
Proportion confirmed (95% CI)^a^	0.929 (0.887, 0.960)	0.631 (0.583, 0.669)	0.298 (0.243, 0.355)	7.69 (4.51, 14.48)	<0.001^b^
≥90% confirmed doses, *N* (%)^c^	32 (78.0)	0 (0.0)	(64.8, 87.0)^a^		<0.001^d^
Total doses confirmed (yes/no)	3,738/284	1,202/702			

^a^Bootstrap estimate.

^b^Wilcoxon rank–sum test.

^c^Patients who have 90% or more of days with confirmed doses.

^d^Fisher’s exact test.

**Abbreviations:** CI, confidence interval; DOT, directly observed therapy; ITT, intent to treat; OR, odds ratio; WOT, wirelessly observed therapy.

**Fig 2:**
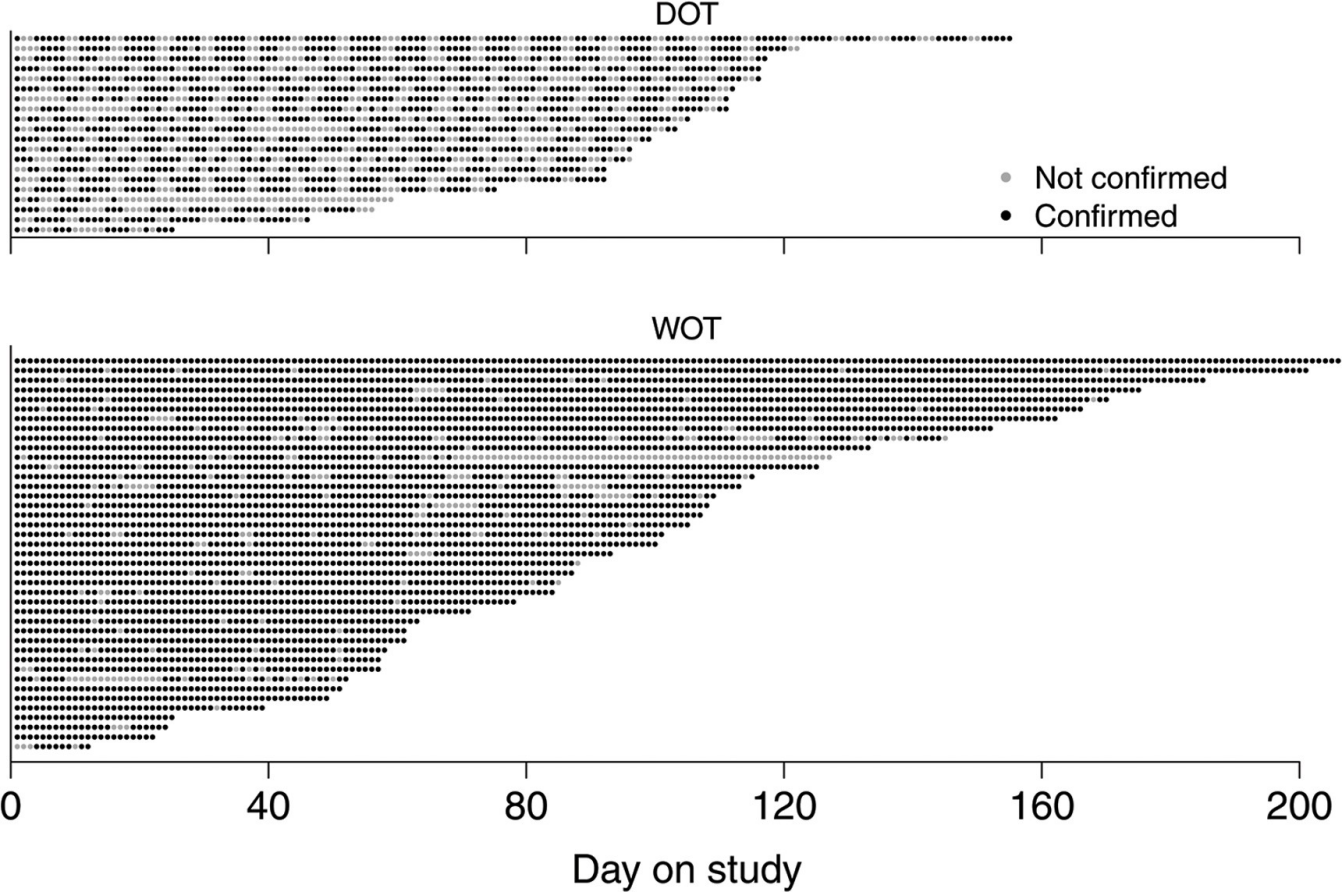
Visualization of confirmed versus unconfirmed doses. Each row represents one patient. Each dot represents one day. Confirmed (black) and not confirmed (gray) doses are shown for each patient on study through the course of the follow-up based on the ITT analysis. Patients are ordered according to their length of follow-up after randomization. DOT, directly observed therapy; ITT, intent to treat; WOT, wirelessly observed therapy.

Fig 2 provides a visualization of confirmed versus unconfirmed doses based on the ITT analysis. A more conservative approach was used to define the confirmed dose as 2 or more pills detected or directly witnessed. This analysis yielded slightly smaller but still statistically significant differences between WOT and DOT arms in proportions of confirmed doses both on the group level (89.2% versus 63.1%; difference = 26.1%; 95% CI = 20.2%, 31.9%; *p* < 0.001) and on an individual level (88.3% versus 61.9%; difference = 26.4%; 95% CI = 19.9%, 32.9%; *p* < 0.001).

To look at the influence of factors on the performance of WOT in comparison to DOT, we performed as-treated analyses incorporating exclusions. Because held doses would have been associated with clinical factors and not whether the participants were receiving WOT or DOT treatment adherence support, these were excluded (Table 4). Using bootstrap estimations, analysis of group level means showed the percent of confirmed doses for WOT was 95.5% (95% CI = 93.5%, 97.1%), in comparison to 63.9% (95% CI 59.0%, 67.8%) for DOT (*p* < 0.001), OR = 11.88. This was very similar to the nonparametric tests for individual-level means: 94.6% (95% CI = 92.4%, 96.8%) in WOT, in comparison to 62.6% (95% CI = 57.0%, 68.3%) for DOT (*p* < 0.001).

**Table 4:** Secondary analysis for comparing proportion of witnessed doses between two treatment arms: days with held doses are excluded, 1 or more tablets are counted as confirmed dose.

	WOT (*N* = 41)	DOT (*N* = 20)	Difference (95% CI)	OR (95% CI)	*p*-Value
Proportion confirmed (95% CI)^a^	0.955 (0.935, 0.971)	0.639 (0.590, 0.678)	0.316 (0.272, 0.368)	11.9 (7.78, 19.5)	<0.001^b^
≥90% confirmed doses, *N* (%)^c^	34 (83.0)	0 (0)	(73.1, 92.5)^a^		<0.001^d^
Total doses confirmed (yes/no)	3,738/178	1,202/680			

^a^Bootstrap estimate.

^b^Wilcoxon rank–sum test.

^c^Patients who have 90% or more of days with confirmed doses.

^d^Fisher’s exact test.

**Abbreviations:** CI, confident interval; DOT, directly observed therapy; OR, odds ratio; WOT, wirelessly observed therapy.

Additional analysis compared the ability of WOT versus DOT to confirm and maintain daily adherence ≥90% in individual participants over the entire period of follow-up (see Tables 3 and 4). The WOT arm contained 78.1% of participants with ≥90% confirmed daily adherence in comparison to 0% in the DOT arm, which was not able to confirm daily adherence (see Table 3). In this ITT analysis, the total missing days of DOT doses were 702 (36.9%); of these, weekends accounted for 538, holidays for 46, held doses for 22, and unspecified for 96. For WOT, a total of 284 (7.1%) doses were recorded as missing; of these, 106 were held. Removing the held doses in the as-treated analysis indicated WOT confirmed daily adherence ≥90% over the entire study period in 83% of participants compared to 0% in the DOT arm, which was unable to confirm daily adherence (see Table 4). While all participants were prescribed daily TB medication, a further secondary analysis evaluated medication adherence but removed all nontraditional working days (that is, all weekends and public holidays) in the DOT arm and all days when medication was held from both arms. In this analysis, the percent of confirmed doses was 95.6% (95% CI 93.6%, 97.2%) in the WOT and 92.7% (95% CI 86.7%, 96.9%) in the DOT arm (*p* = 0.31). The difference between WOT and DOT is 2.8%, 95% CI (−1.8%, 9.1%), indicating non-inferiority of WOT compared to DOT dose confirmation at a very conservative non-inferiority margin of 2% (see S1 Table). Finally, secondary regression analyses of adherence over time, to evaluate evidence of potential treatment fatigue associated with WOT use, indicated that once held doses (independent of treatment arm) were excluded, there was no difference in adherence over time between arms, despite a significantly longer follow-up period for participants on WOT.

### Adverse events and participant experience

In terms of adverse events, there was no significant difference in the rate of adverse events between the WOT and DOT arms. Adverse events observed in the WOT arm that could be related to WOT use occurred in 9.8% of participants; almost all constituted mild grade 1 reactions to the patch, either mild redness or itching, with the exception of a participant who reported pruritis, which was judged as grade 2 moderate severity. These events were easily mitigated by moving the patch to a different location on the torso and did not interrupt WOT use or contribute to nonadherence in the WOT arm. One participant withdrew from Stage 1 and none withdrew from Stage 2 of the randomized study because of skin irritation associated with the monitor patch. No adverse events associated with the sensor ingestion were observed.

All participants (100%) stated they would prefer to continue to use WOT and not DOT at randomization. During the study, the public health department requested that participants be allowed to continue on WOT until the completion of their treatment because of participants’ reluctance to return to SOC DOT. In terms of ease of use, 75.3% in Stage 1 and 92.8% in Stage 2 reported being comfortable replacing the patch on their own, and 39.4% in Stage 1 and 31% in Stage 2 reported the most difficult step associated with this was choosing a location on the body to place the new patch. When asked what could be done to improve the WOT system, the most common replies were either “nothing” or “make the patch smaller.” Other common responses were “removable patch between doses,” “ability to switch side of patch location,” “better adhesive/glue,” and “better adhesive for sensitive skin.”

### Longitudinal dosing patterns visualized by WOT

**Fig 3:**
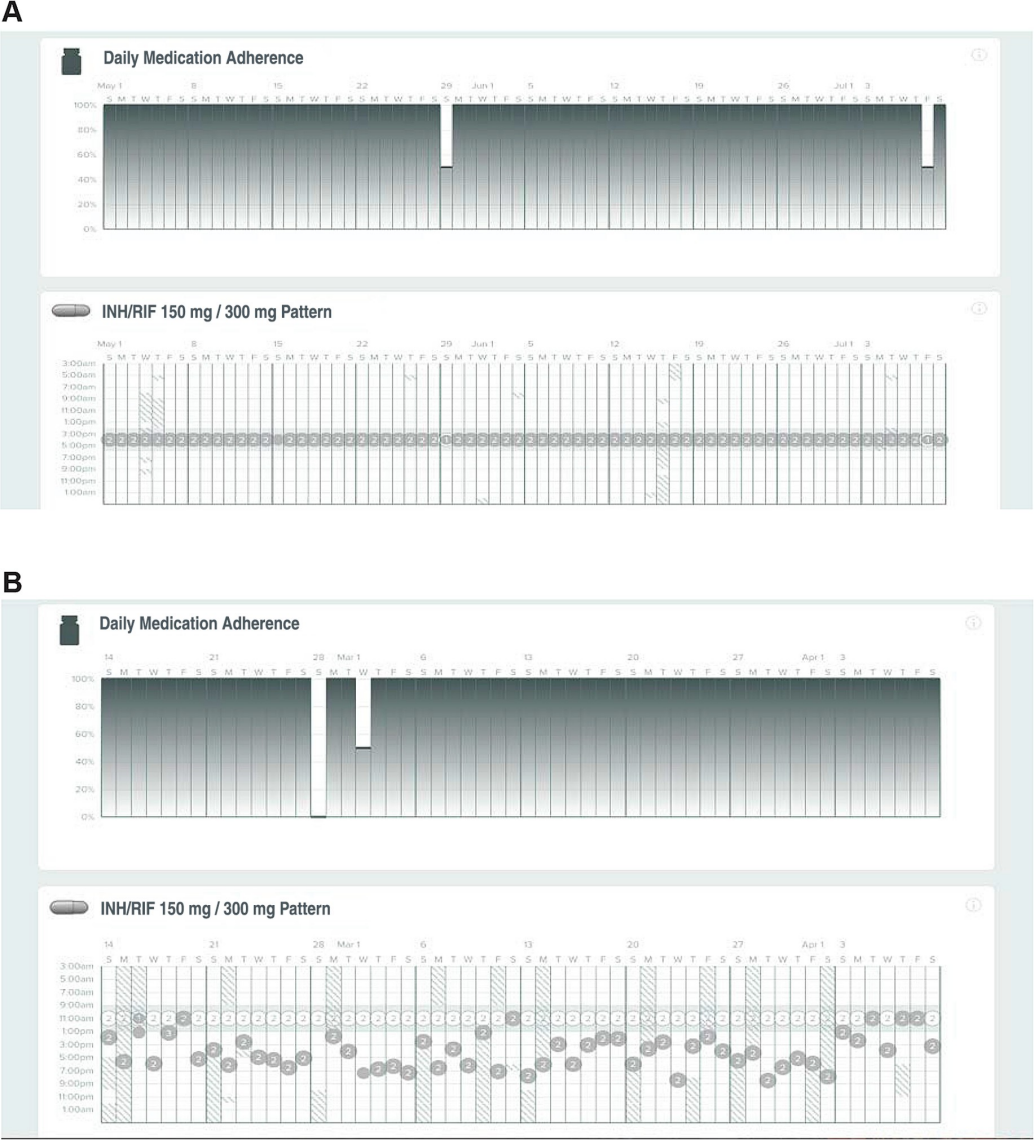
Longitudinal patterns of taking and timing adherence data of IS-Rifamate using WOT in two participants. The first participant has highly regular taking and timing adherence, in this case achieved by setting an alarm reminder. The second participant displays variable timing adherence, but taking adherence is maintained with WOT adherence support. INH, isoniazid; IS, ingestion sensor; IS-Rifamate, IS-enabled combination isoniazid 150 mg/rifampin 300 mg; RIF, rifampin; WOT, wirelessly observed therapy.

The ability to provide data on longitudinal patterns of medication ingestion remotely may allow differential support to patients who need it, improving the utilization of limited public health resources. Fig 3 shows the ability of the WOT system to provide detailed visual summaries of adherence data based on ingestion. Such visualizations were generated for each participant and updated daily. The dates are represented along with the time of ingestion and the number of pills ingested. Trace A shows the record of a participant who had highly regular medication taking and timing, whereas Trace B shows the record of a participant whose medication-taking profile was more erratic and required reminders to maintain adherence.

## Discussion

DOT is the current highest standard to confirm oral medication taking in TB treatment. The PDA of WOT, determined by the number of ingestions confirmed by WOT conducted under simultaneous direct observation, was extremely high in this study: 99.3% (CI 95%: 98.1%, 100%). These data were obtained from patients with active TB in clinical and home settings, using primarily county outreach workers, and represent the accuracy and value of the WOT system when used clinically, not in idealized laboratory circumstances. In terms of accuracy, WOT was equivalent to DOT within US public health TB programs and provided digital documentation of medication taking. This comparison of WOT and DOT has policy implications for TB treatment within the US. If recommendations for TB treatment require DOT dosing, based on our results, a WOT dose can be considered equivalent to a DOT dose. Our findings provide rigorous data supporting WOT accuracy in clinical and home settings during the continuation phase of TB treatment.

WOT is the first sensor-based system to confirm medication ingestion; its components (patch and IS) are FDA-approved, and the system is the only technology approved by the FDA for the measurement of oral medication adherence [38]. This wireless-sensor–based technology has the potential to be superior to DOT on a number of levels. WOT confirms medication ingestion, whereas DOT does not guarantee actual ingestion has taken place because patients may hide pills in their mouths [39–42]; WOT provides immediate digital record of medication ingestion, whereas DOT relies largely on written verification; WOT operates daily, whereas DOT does not in public health departments. As reviewed in our introduction, attempts to reduce DOT below 5 days a week because of the complexity of DOT implementation and cost have resulted in dosing distortions associated with outcomes clearly inferior to daily dosing [14, 15]. The ubiquity of cell phone technology, with its ability to transfer information over both wireless and cellular networks, means that WOT use does not require highly developed public health infrastructures with large numbers of personnel, as are required for DOT, and that the numbers of individuals WOT can follow is essentially unlimited. Because WOT has the potential to provide a new, rigorous approach for documenting and supporting daily medication adherence in TB treatment, it was critical for us to perform a randomized trial of WOT compared to DOT within US public health departments utilizing well-funded, successful DOT programs. In so doing, we provide comparison of WOT to the highest standard of in-person DOT available for TB treatment adherence support and reporting.

Results from our randomized trial in such settings provide evidence that WOT is able to support daily medication adherence in TB treatment and that it confirmed significantly more doses in the ITT analysis than SOC DOT. The SOC DOT comparator arm was performed in highly funded southern Californian TB control programs. As expected, sensitivity analysis with all nonworking days and held doses removed from each arm showed WOT was non-inferior to DOT. However, the ITT and sensitivity analyses indicate that the comparator arm SOC DOT was not available for approximately 30% of daily prescribed TB medication doses. Because daily medication taking is important to maintain adequate bacteriostatic and bactericidal activities of INH and rifampin during TB treatment, the design of this study aimed to provide data on the vital question of how well WOT technology operated daily in comparison to maximal DOT. WOT in this trial utilized the near-real–time data transfer to identify daily ingestion in patient participants. The technology was able to clearly identify individual patterns of medication adherence, with clear, easy-to-interpret visualizations providing healthcare workers with actionable information they could review in seconds (see Fig 3). Very importantly from a resource utilization perspective, WOT enabled differentiated patient care, allowing direction of personnel resources to patients who needed support to maintain medication adherence. Because the data are digital, there was no waiting time for the completion of documentation, a notable distinction from the DOT written records, which were frequently passaged though multiple personnel and, in some cases, took weeks to reach the medical chart.

Our trial confirmed WOT was safe, with side effects limited to skin irritation associated with wearing the patch, and was easy to use. None of the randomized participants in this trial wanted to be returned to DOT. The majority of our study population were well below the federal poverty line and did not have advanced education or English as their first language. WOT was developed as a patient self-management system, and our study confirms the system can be initiated and maintained in patients without requiring extensive healthcare worker input. All the randomized participants wanted to continue using the WOT system, and 100% of participants stated they preferred WOT to DOT. WOT represents a “stand-alone” digital, self-managed medication adherence support system in which all data are digitally stored in near real time. WOT does not require secondary interpretation or large data file transfers, is available remotely online via a website accessed with patient permission, and can produce instantaneous digital summary records and analytics, and these digital adherence data can be directly placed into electronic medical records. Since this study, the WOT system has evolved and improved. Current WOT utilizes a “Bring Your Own Device” smartphone system (Android or Apple) and a newer, adhesive patch changed every 5–7 days with a single reusable detector hub. The technology does have the capacity to be used with standard-feature cell phones, but this is not yet available.

This trial has a number of limitations, which include the collection of data only during the continuation phase of TB therapy. Sensors within the WOT system are uniquely coded to individual tablet identity and strength. The sensors are independent of gastric PH, gastric structure, or concomitant medications. Currently available data indicate that 6–10 sensors swallowed simultaneously can be detected [40]. This is important because during the intensive phase of TB treatment and in novel oral MDR regimens, patients take multiple medications of different strengths, and thus, the numbers of tablets taken at one time may be high. Data are needed on the performance of WOT during intensive-phase TB treatment, and evaluation of this is underway in southern California. In addition, DOT in this trial was optimized because no one receiving twice- or three-times-a-week DOT was included, and the DOT programs were highly staffed.

Globally, it is critical that WOT be tested in high-burden TB settings. Global settings have quickly utilized cell-phone–based communication structures in areas where infrastructure development is lacking. A notable example of this is the adoption of bill pay, banking, and management of businesses using such networks in developing countries, which significantly predates that in developed countries. WOT involves small amounts of data transfer by current standards, can work in lower-speed internet and cellular network environments, and, as stated above, is capable, with adaptation, of being used with standard-feature phones. Moreover, the ability of WOT to provide immediate digital treatment records, maintained by FDA-regulated private industry with the highest standards of encryption and constantly upgraded user-friendly software, could substantially support TB programs in middle- and low-income settings, which largely operate off inadequate paper record systems.

Key medication changes are now in transition for MDR-TB regimens that would enable the first entirely oral treatment regimen [3]. Major treatment principles espoused in these recommendations are social support to enable adherence to treatment to ensure a patient-centered approach to care and drug safety management and monitoring. Our research demonstrates WOT is a self-management system that can provide near-real–time actionable information, allowing patient-centered care to support adherence. WOT has the capability of monitoring multiple drugs within complex regimens individually. In addition, because WOT also monitors physiological measures, it has the capacity to identify changes in QT intervals, a significant issue with moxifloxacin/levoquin, bedaquiline, and clofazimine in Group A and B oral MDR regimens. It is vital that medication adherence support be incorporated in the use of novel oral MDR regimens. The findings of this study suggest that WOT technology offers advantages over DOT for adherence confirmation and support and that WOT should be incorporated into implementation trials of oral MDR regimens in global settings.

## Supporting information

S1 CONSORT Checklist This file contains the CONSORT checklist indicating where in the paper each CONSORT element is located. CONSORT, Consolidated Standards of Reporting Trials.
(DOC)

S1 Fig Ingestible sensor. Photo courtesy of TallGrass Pictures, San Diego, CA.
(TIF)

S2 Fig Patient self-management system and information flow within the developed WOT system. 1) At home, the patient takes the digitized medicine. The IS activates in the stomach, and its serial number is captured and stored by the patch. 2) Patch data are transferred by Bluetooth to an app on the patient’s mobile device. 3) Patients can follow their own medication taking and receive automated reminders. 4) Data are transferred to secure servers. 5) This enables patients to share their medication taking behavior with others in their social support network if they choose. 6) In addition, patient-approved healthcare workers can remotely monitor and confirm TB treatment adherence, providing timely support as needed, to large cohorts of patients using the secure web-based dashboard. Support or intervention if needed can be provided in a highly targeted manner (images of the person and those labeled 1, 2, and 6 were provided courtesy of Proteus Digital Health). IS, ingestion sensor; TB, tuberculosis; WOT, wirelessly observed therapy.
(TIF)

S1 Table Proportion of confirmed doses, WOT versus DOT, with all weekend days and public holidays excluded in DOT arm, and days with “held” doses excluded in both arms. Confirmed dose defined as 1 or more tablets detected or witnessed. DOT, directly observed therapy; WOT, wirelessly observed therapy.
(PDF)

S1 Text Research protocol. WOT in comparison to DOT for the treatment of TB. DOT, directly observed therapy; TB, tuberculosis; WOT, wirelessly observed therapy.
(PDF)

## Abbreviations

ANC: absolute neutrophil count
AVRC: Anti-Viral Research Center
CBC: complete blood count
CI: confidence interval
CMP: comprehensive panel
CONSORT: Consolidated Standards of Reporting Trials
DOT: directly observed therapy
DOTS: short-course directly observed therapy
GED: general education development
HS: high school
IGRA: interferon gamma release assay
IQR: interquartile range
IS: ingestion sensor
IS-Rifamate: IS-enabled combination isoniazid 150 mg/rifampin 300 mg
ITT: intent to treat
LMIC: low- and middle-income country
MDR-TB: multidrug-resistant tuberculosis
OC: Orange County
OR: odds ratio
PDA: positive detection accuracy
PK: pharmacokinetic
RCT: randomized control trial
SAT: self-administration therapy
SD: San Diego
SOC: standard of care
TB: tuberculosis
UCSD: University of California San Diego
VOT: video observed therapy
WOT: wirelessly observed therap
