# Supplementary figures and images for: Wirelessly observed therapy compared to directly observed therapy to confirm and support tuberculosis treatment adherence: A randomized controlled trial
Source: PLoS Med. 2019 Oct 4;16(10):e1002891. doi: 10.1371/journal.pmed.1002891 (PMC6777756; doi:10.1371/journal.pmed.1002891)

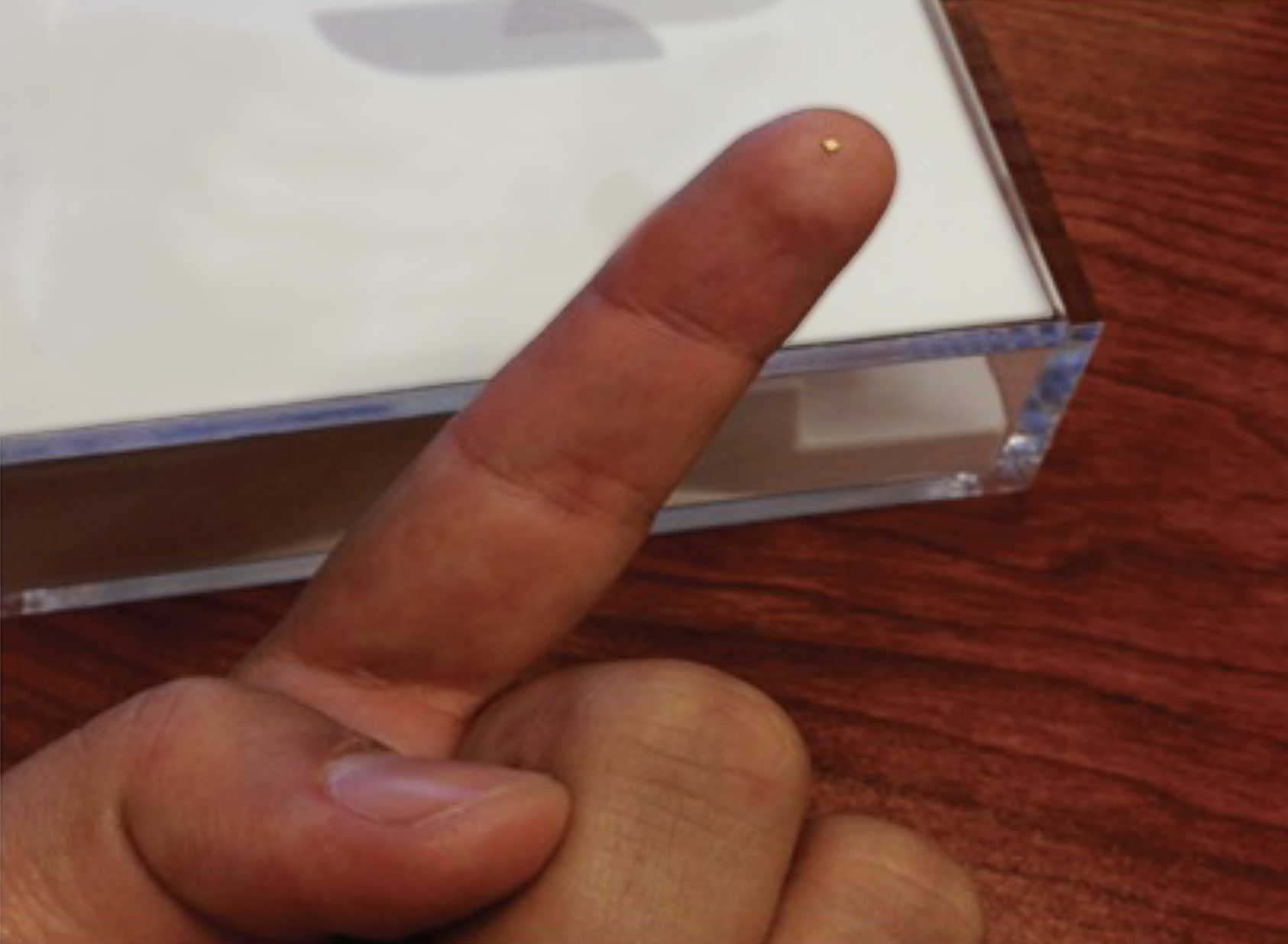

Supplement: S1 Fig — Photo courtesy of TallGrass Pictures, San Diego, CA. (TIF) [file pmed.1002891.s002.tif]

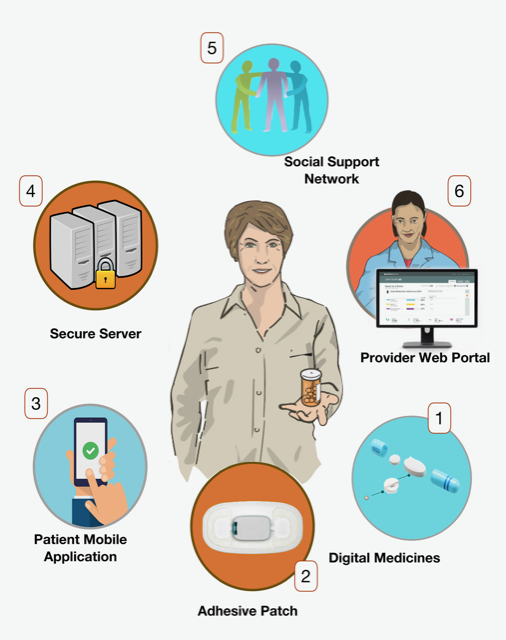

Supplement: S2 Fig — 1) At home, the patient takes the digitized medicine. The IS activates in the stomach, and its serial number is captured and stored by the patch. 2) Patch data are transferred by Bluetooth to an app on the patient’s mobile device. 3) Patients can follow their own medication taking and receive automated reminders. 4) Data are transferred to secure servers. 5) This enables patients to share their medication taking behavior with others in their social support network if they choose. 6) In addition, patient-approved healthcare workers can remotely monitor and confirm TB treatment adherence, providing timely support as needed, to large cohorts of patients using the secure web-based dashboard. Support or intervention if needed can be provided in a highly targeted manner (images of the person and those labeled 1, 2, and 6 were provided courtesy of Proteus Digital Health). IS, ingestion sensor; TB, tuberculosis; WOT, wirelessly observed therapy. (TIF) [file pmed.1002891.s003.tif]
