# Supplementary material for: Wirelessly observed therapy compared to directly observed therapy to confirm and support tuberculosis treatment adherence: A randomized controlled trial
Source: PLoS Med. 2019 Oct 4;16(10):e1002891. doi: 10.1371/journal.pmed.1002891 (PMC6777756; doi:10.1371/journal.pmed.1002891)
Supplement: S1 Table — Confirmed dose defined as 1 or more tablets detected or witnessed. DOT, directly observed therapy; WOT, wirelessly observed therapy. (PDF) [file pmed.1002891.s004.pdf]

Supplementary Table 1: *Proportion of confirmed doses, WOT vs. DOT, with all weekend days and public holidays excluded in DOT arm, and days with 'held' doses excluded in both arms. Confirmed dose defined as 1 or more tablets detected or witnessed.*

	WOT (N=41)	DOT (N=20)	Difference (95% CI)	Odds Ratio (95% CI)	P-value
Proportion confirmed (95% CI) [a]	0.956 (0.936, 0.972)	0.927 (0.867, 0.969)	0.028 (-0.018, 0.091)	1.68 (0.64, 3.86)	0.31 [c]
≥90% confirmed doses, N (%) [b]	34 (82.9)	16 (80.0)	(-7.8, 26.0) [a]		1.00 [d]
Total doses confirmed (yes/no)	3738/174	1202/94			

Notes: [a] Bootstrap estimate; [b] patients who have 90% or more of days with confirmed doses; [c] Wilcoxon rank sum test; [d] Fisher's exact test. CI = confidence interval
