# Supplementary material for: Wirelessly observed therapy compared to directly observed therapy to confirm and support tuberculosis treatment adherence: A randomized controlled trial
Source: PLoS Med. 2019 Oct 4;16(10):e1002891. doi: 10.1371/journal.pmed.1002891 (PMC6777756; doi:10.1371/journal.pmed.1002891)
Supplement: S1 Text — WOT in comparison to DOT for the treatment of TB. DOT, directly observed therapy; TB, tuberculosis; WOT, wirelessly observed therapy. (PDF) [file pmed.1002891.s005.pdf]

AHF TB 001

Wirelessly Observed Therapy in Comparison to Directly Observed Therapy for the Treatment of Tuberculosis

A Pilot Clinical Trial Characterizing Use of Ingestion Sensor Enabled Rifamate in Comparison to Directly Observed Therapy for the Treatment of Tuberculosis

A Clinical Trial Funded by:
The Alliance Healthcare Foundation & Specialists in Global Health

Technology Provided by:
Proteus Digital Health, Inc.

Protocol Chair: Sara H. Browne, MD, MPH

Version 2.3
March 25, 2015

CONTENTS

PROTOCOL TEAM ROSTER **4**

ABBREVIATIONS USED **6**

SITES PARTICIPATING IN THE STUDY **8**

SCHEMA **10**

1.0 HYPOTHESIS AND STUDY OBJECTIVES **12**
1.1 Hypotheses 12
1.2 Study Objectives 12

2.0 INTRODUCTION **13**
2.1 Background 13
2.2 Rationale 14

3.0 STUDY DESIGN **17**
3.1 Non-Significant Risk Study 18
3.2 Study Population 18
3.3 Recruitment 19
3.4 Study Conduct and Data Collection 20
3.5 Study Design 20
3.6 Study Outcome Data 24
3.7 Discontinuation for Medication Related Toxicity 24
3.8 Plasma Storage 24

4.0 SELECTION AND ENROLLMENT OF SUBJECTS **25**
4.1 Inclusion Criteria 25
4.2 Exclusion Criteria 25
4.3 Study Enrollment Procedures 26

5.0 STUDY TREATMENT **26**
5.1 The Ingestible Sensor System 26
5.2 Regimens, Administration, and Duration 26
5.3 Study Treatment (DHFS IS-RM) Formulation and Preparation 27
5.4 Study Product Supply, Distribution, and Accountability 28
5.5 Concomitant Medications 28

6.0 CLINICAL AND LABORATORY EVALUATIONS **31**
6.1 Schedule of Events 31
6.2 Evaluations 33
6.3 Special Instructions and Definitions of Evaluations 36
6.4 Off-Drug Requirements 38

7.0 CLINICAL MANAGEMENT ISSUES 39
7.1 Adverse Events 39
7.2 Toxicity Management 41

8.0 CRITERIA FOR DISCONTINUATION 43
8.1 Criteria for Treatment Discontinuation 43
8.2 Criteria for Discontinuation from the Study 43

9.0 STATISTICAL CONSIDERATIONS 44
9.1 General Design Issues 44
9.2 Endpoints 44
9.3 Randomization 45
9.4 Sample Size and Accrual 45
9.5 Monitoring 46
9.6 Analyses 46

10.0 PHARMACOLOGY PLAN INCLUDING INTENSIVE PK SUBSTUDY 49
10.1 Pharmacology Substudy Objectives 49
10.2 Pharmacology Substudy Design 49
10.3 Primary and Secondary Data, Modeling, and Data Analysis 52
10.4 Preliminary Results 52

11.0 DATA COLLECTION AND MONITORING AND ADVERSE EXPERIENCE REPORTING 52
11.1 Records to Be Kept 52
11.2 Project Management by BIT Core 56
11.3 Role of Data Management 57
11.4 Clinical Site Monitoring and Record Availability 57
11.5 Serious Adverse Experience Reporting 57

12.0 HUMAN SUBJECTS 57
12.1 Institutional Review Board Review and Informed Consent 57
12.2 Subject Confidentiality 58
12.3 Study Discontinuation 58

13.0 PUBLICATION OF RESEARCH FINDINGS 58

14.0 BIOHAZARD CONTAINMENT 58

PROTOCOL TEAM ROSTER

Chair
Sara H. Browne, MD, MPH
Assoc. Professor of Clinical Medicine,
Div. of Infectious Diseases,
George Palade Labs, Rm 125
University of California, San Diego
9500 Gilman Drive
La Jolla, CA 92093
[redacted]

Co-Investigators:
Constance A. Benson, MD
Professor of Medicine
Division of Infectious Diseases
I.D. Training Program Director
Director, Antiviral Research Center
PI/Director, HIV/AIDS Clinical Trials Unit
University of California, San Diego
220 Dickinson Street, Suite A
San Diego, CA 92103
[redacted]

Kathleen S. Moser, MD
Public Health Services
San Diego County HHSA
P.O. Box 85222 (P511D)
San Diego, CA 92186
[redacted]

Julie Low MD
County of Orange Health Care Agency
Pulmonary Disease Services
1725 W 17th Street,
Santa Ana, CA 92706
[redacted]

Terrence F. Blaschke,
Emeritus Professor of Medicine and
Pharmacology, Stanford University,
291 Campus Drive,
Stanford,
CA 94305
[redacted]

Charles Peloquin, Pharm.D.
College of Pharmacy
University of Florida
1600 SW Archer Road, P4-33
Gainesville, FL 32610
[redacted]

Project Management:
Amanda Tucker Maytom, MD
University of California, San Diego
Antiviral Research Center
220 Dickinson Street, Suite A
San Diego, CA 92103
[redacted]

Study Co-ordinator:
Jonathan Gonzalez-Garcia
Staff Research Associate
University of California, San Diego
Antiviral Research Center
220 Dickinson Street, Suite A
San Diego, CA 92103
[redacted]

Study Nurse
Jill Kunkel, RN
University of California, San Diego
Antiviral Research Center
220 Dickinson Street, Suite A
San Diego, CA 92103
[redacted]

Data Management
Amanda Tucker Maytom, MD

University of California, San Diego
Antiviral Research Center
220 Dickinson Street, Suite A
San Diego, CA 92103
[redacted]

Anya Umlauf, PhD
Senior Statistician,
HNRC, Rm 344,
220 Dickinson Street,
San Diego CA 92103
[redacted]

<u>Statisticians</u>

Anya Umlauf, PhD
Senior Statistician,
HNRC, Rm 344,
220 Dickinson Street,
San Diego CA 92103
[redacted]

Florin Vaida, PhD
Prof. Family Medicine & Public Health
University of California, San Diego
9500 Gilman Drive,
La Jolla, CA 92093
[redacted]

<u>Pharmacokinetic Analysis</u>

Charles Peloquin, Pharm.D.
College of Pharmacy
University of Florida
1600 SW Archer Road, P4-33
Gainesville, FL 32610
[redacted]

[redacted]

<u>Pharmacist</u>

Leticia Muttera
Investigational Pharmacist
University of California, San Diego
Antiviral Research Center
220 Dickinson Street, Suite A
San Diego CA 92103
[redacted]

<u>Laboratory Technologist</u>

DeeDee Pacheco
Phlebotomist and Lab Manager
University of California, San Diego
Antiviral Research Center
220 Dickinson Street, Suite A
San Diego, CA 92103
[redacted]

<u>Industry Representative:</u>

George Savage, MD
Chief Medical Officer,
Proteus Digital Health, Inc.
2600 Bridge Parkway, Suite 101
Redwood City, CA 94065
[redacted]

ABBREVIATIONS USED

AAE	anticipated adverse event
ACTG	AIDS Clinical Trials Group
AE	adverse event
AFB	acid-fast bacilli
AHF	Alliance Healthcare Foundation
ALT	alanine transaminase
ANC	absolute neutrophil count
AST	aspartate transaminase
AUDIT	Alcohol Use Disorders Identification Test
AVRC	Antiviral Research Center
BIT Core	UCSD Center for AIDS Research Bioinformatics and Information Technologies Core
BUN	blood urea nitrogen
CBC	complete blood count
CDC	Centers for Disease Control and Prevention
CMD	Core Medical Device
CMP	comprehensive panel
DAST-10	Drug Abuse Screening Test
DHFS	digital health feedback system
DOT	directly observed therapy
FDA	U.S. Food and Drug Administration
β-HCG	human chorionic gonadotropin
HLA	human leukocyte antigen
HHSA	County of San Diego Health and Human Services Agency
HIV	human immunodeficiency virus
IEM	ingestible event markers
IRB	institutional review board
IS-RM	ingestion sensor-enabled Rifamate
MDR-TB	multi-drug-resistant tuberculosis
NIH	National Institutes of Health
OCCAMS	Open Source Clinical Content Management System
OCHCA	County of Orange Health Care Agency Pulmonary Disease Services
PDA	positive detection accuracy
PDH	Proteus Digital Health
PHI	protected health information
PHQ-9	Patient Health Questionnaire-9
PHS	County of San Diego Public Health Services
PI	Principal Investigator
QD	once a day
SAE	serious adverse event
SAP	statistical analysis plan
SGOT	serum glutamic oxaloacetic transaminase
SGPT	serum glutamic pyruvic transaminase
SOC	standard of care
TB	Mycobacterium tuberculosis complex

UA	urinalysis
UADE	Unanticipated Adverse Device Effect
UCSD	University of California, San Diego
ULN	upper limit of normal
USP	United States Pharmacopeia
WHO	World Health Organization
WOT	wirelessly observed therapy

SITES PARTICIPATING IN THE STUDY

UCSD Antiviral Research Center

The University of California, San Diego (UCSD) is among the world's leading institutions for HIV patient care and clinical, basic, and behavioral HIV research. The UCSD Antiviral Research Center (AVRC), founded in 1986 by Drs. Douglas Richman, Stephen Spector, and Allen McCutchan, has been at the forefront of clinical training and research since the beginning of the HIV epidemic in the U.S. The AVRC is engaged in multiple studies funded by the National Institutes of Health (NIH), including trials with the AIDS Clinical Trials Group (ACTG), the Centers for Disease Control (CDC), and California Research Programs. This highly distinguished center has performed investigational clinical trials with most of major international pharmaceutical companies including Merck, Pfizer, GlaxoSmithKline, Gilead, Bristol-Myers Squibb, Tibotec, Roche, Abbott, Boehringer Ingelheim, Vertex, Dupont, and Egron. The AVRC participates in approximately 60 clinical trials per year.

AVRC researchers have also been active in clinical trials related to Mycobacterium tuberculosis complex (TB) infection with the Tuberculosis Trials Consortium, the CDC, and the ACTG. Current TB trials at the AVRC include studies of pulmonary disease in TB infected subjects (with and without HIV), pharmacokinetics of higher doses of standard treatments for TB, and cross border TB infection. In partnership with the CDC, County of San Diego Health and Human Services Agency (HHSA), and the University of California, San Francisco, the AVRC is currently running a study to assess the relative performance and cost of three U.S. Food and Drug Administration (FDA)-approved diagnostic tests for latent TB infection. In addition, the AVRC collaborates with the La Jolla Institute for Allergy and Immunology and the San Diego Blood Bank in an NIH study to identify, characterize, and validate human leukocyte antigen (HLA) class II epitopes derived from Mycobacterium tuberculosis. In collaboration with the HHSA, investigators at the AVRC have completed a proof of concept study on use of video directly observed therapy (DOT) in TB, a novel mobile technology.

San Diego County Public Health

County of San Diego Public Health Services (PHS) is dedicated to community wellness and health protection for the people of San Diego County. The PHS, part of the HHSA, works to prevent epidemics and the spread of disease, protect against environmental hazards, prevent injuries, promote and encourage healthy behaviors, respond to disasters, and assist communities in recovery and assure the quality and accessibility of health services throughout the county. Diagnostic and treatment services for TB are provided at the main TB Clinic, located at the Health Services Complex on Rosecrans Street in San Diego. The AVRC and HHSA have a long track record of collaborating on clinical studies of TB within San Diego County.

County of Orange Health Care Agency Pulmonary Disease Services

County of Orange Health Care Agency (OCHCA) is dedicated to community wellness and health protection for the people of Orange County. The OCHCA works to prevent epidemics and the spread of disease, protect against environmental hazards, prevent injuries, promote and encourage healthy behaviors, respond to disasters, and assist communities in recovery and assure

the quality and accessibility of health services throughout the county. Orange County TB Control is housed within the Pulmonary Disease Services Program. TB screening, diagnosis, and treatment services are offered to Orange County residents at the TB Treatment and Prevention Services Clinic on West 17th Street in Santa Ana.

SCHEMA

AHF TB 001

Wirelessly Observed Therapy in Comparison to Directly Observed Therapy for the Treatment of Tuberculosis

A pilot feasibility trial characterizing use of a digital health feedback system (DHFS) using ingestion sensor-enabled fixed-dose combination of isoniazid-rifampin (Rifamate) and comparing this to directly observed therapy (DOT) for treatment of Mycobacterium tuberculosis complex (TB).

Design: AHF TB 001 is a multi-site, randomized, open-label, controlled interventional pilot feasibility study comparing the efficacy, characteristics of use, persistence and safety of a digital health feedback system (DHFS) (Proteus Digital Health, Inc., Redwood City, CA, USA) using ingestion sensor-enabled fixed-dose combination Rifamate (IS-RM) in comparison to directly observed therapy (DOT) using standard of care (SOC) with isoniazid plus rifampin, or Rifamate in TB-infected subjects. Subjects will be ineligible if they have any evidence of drug resistant TB or prior intolerance to study drug. Those who are found to be eligible will participate in a two-week investigation (Phase 1) of the positive detection accuracy and characteristics of use of the DHFS in the TB patient population. Eligible patients who elect to continue in Phase 2 will then be randomized 2:1 to monitoring intervention with the ingestion sensor-enabled Rifamate (IS-RM), or to DOT SOC. This study will be conducted at the UCSD AVRC, the San Diego County TB Clinic, and the County of Orange Health Care Agency Pulmonary Services Clinic.

Duration: Up to 12 months

Sample Size: 75-100 subjects in Phase 1; 50 intervention and 25 control SOC subjects in Phase 2

Study Population: Adult men and women being treated for TB with at least isoniazid and rifampin who are able to use the DHFS mobile device, and to tolerate a wearable sensor patch

Regimen: Subjects will be randomized 2:1 to TB treatment using DHFS ingestion sensor-enabled Rifamate (IS-RM) or SOC

Figure 1. AHF TB 001 Study Schema

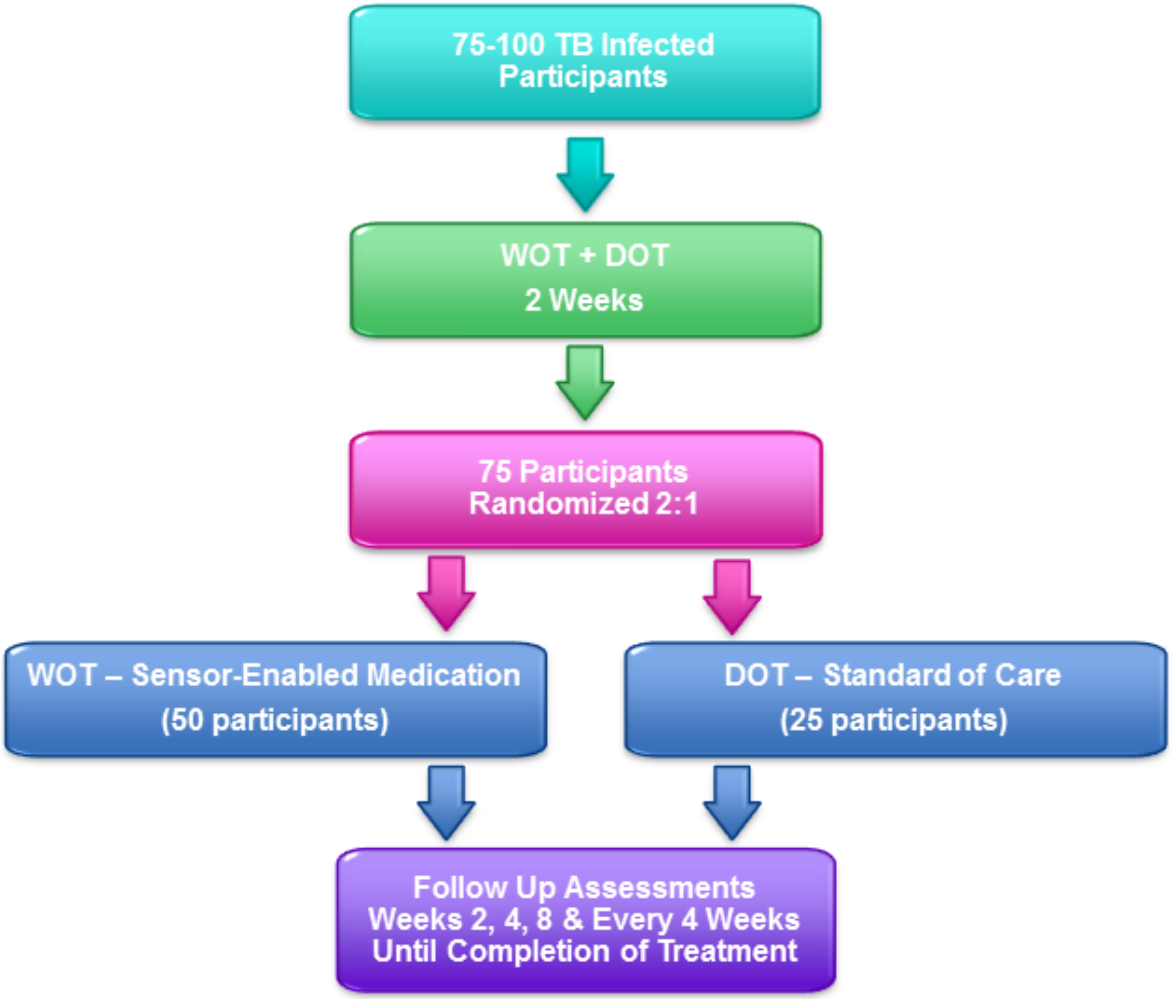

1.0 HYPOTHESIS AND STUDY OBJECTIVES

1.1 Hypotheses

1.1.1 Direct confirmation of TB medication dosing by DHFS is feasible for monitoring TB treatment.

1.1.2 DHFS can be used as an alternative to SOC DOT for monitoring and documenting completion of TB treatment.

1.1.3 The cost of TB target treatment using DHFS is comparable or favorable when compared to DOT for monitoring a comparable number of targeted doses.

1.1.4 DHFS is safe for use in TB treatment.

1.1.5 Patient satisfaction with DHFS use for TB treatment is rated as 'satisfactory' or higher.

1.1.6 Healthcare worker and physician satisfaction with DHFS for TB treatment is rated as 'satisfactory' or higher.

1.1.7 DHFS subjects have similar or better self-efficacy scores than control subjects receiving SOC DOT during and at the end of the trial.

1.2 Study Objectives

Primary

1.2.1 Phase 1: Determine positive detection accuracy (PDA, direct confirmation of TB medication ingestion) of the DHFS when compared to a healthcare worker witnessing actual TB medication ingestion. PDA will be based upon proper DHFS use. PDA will be determined using an intention-to-treat and an adjusted analysis.

1.2.2 Phase 2: Determine the percentage of witnessed doses by DHFS and SOC, respectively. Witnessed doses by DHFS is defined as the total number of dose ingestions confirmed by DHFS, divided by the total number of prescribed witnessed dose ingestions for TB treatment. Witnessed doses by SOC is defined as the total number of dose ingestions that are actually witnessed by a healthcare worker, divided by the total number of prescribed witnessed dose ingestions for TB treatment.

Secondary

1.2.3 Determine the percentage of subjects completing > 80% prescribed witnessed dosing for TB treatment using DHFS and SOC, respectively.

1.2.4 Summarize the total number of lapses in witnessed doses and longest duration of witnessed treatment lapse during use of DHFS and SOC, respectively.

1.2.5 Model cost of treatment based upon data obtained during use of DHFS and SOC, respectively.

1.2.6 Characterize adverse events for DHFS and SOC using summary statistics.

1.2.7 Characterize subject responses to post-study questionnaires to collect information regarding their experience with the DHFS and the usability of the system, using summary statistics. Questionnaire responses by subjects will be examined by study reviewers from the AVRC to develop recommendations regarding the usability of the system.

1.2.8 Characterize provider responses to post-study questionnaires to collect information regarding their experience with the DHFS and the usability of the system, using summary statistics. Questionnaire responses by providers will be examined by study reviewers from the AVRC to develop recommendations regarding the usability of the system.

1.2.9 Characterize generalized scores on habitual self-control, self-efficacy beliefs, depression scale (PHQ-9), alcohol use (AUDIT), and drug use (DAST-10) questionnaires at the start and end of study within the intervention group, and between the intervention and control groups.

1.2.10 Define the characteristics of those subjects with < 80% adherence, including demographics, disease characteristics, and self-reported metrics by individual instruments, for example the PHQ-9.

2.0 INTRODUCTION

2.1 Background

Eradication of disease due to *Mycobacterium tuberculosis complex* (TB) has been for decades a major policy goal of the Centers for Disease Control (CDC), the Global Alliance for TB Drug Development, and the World Health Organization (WHO). Poor adherence to therapy has long been recognized as a barrier to effective treatment of TB infection. Inconsistent and interrupted treatment, in combination with HIV co-infection, has led to rising numbers of TB cases and the emergence of drug resistant TB. One third of the world's population (~2 billion people) are infected with latent TB, over 9 million people a year develop active TB, and mortality approaches 2 million deaths annually.

Treatment of active TB requires 6-12 months of combined therapy. Public health authorities in all countries recommend that TB treatment be administered by short course directly observed therapy (DOT), in which a healthcare worker observes medication ingestion events and provides written verification of treatment adherence and completion. While highly reliable when performed appropriately, DOT is resource intensive, time consuming, and represents the largest

single cost of TB treatment [1]. Although DOT is the WHO's recommended TB treatment standard, its implementation has been attenuated due to cost. Even in the U.S., DOT is not consistently applied; "modified DOT," or self-administered therapy, is more often used due to budget limitations. Some TB public health programs in the U.S. do not use DOT at all, or use DOT only for high-risk patients; in the private sector, self-administered therapy is the rule rather than the exception. Bi- and tri-weekly dosing is used to lower the cost of DOT, despite evidence that daily dosing of TB medication is more effective [2, 3]. In the absence of DOT, there is no method of reliably determining when and if patients actually ingest their medication.

Alternative methods for assessing adherence such as patient questionnaires, pill counts, patient diaries, and prescription refill rates have been shown to be inaccurate and to overestimate adherence [4-6]. For example, a review of prescription databases can show when refills occur, but does not show when dosing errors occurred, or most importantly, the date the patient stopped taking the drug [7-10]. Limitations also apply to measurements of drug concentrations in plasma, which do not take into account the fact that patients' drug taking behavior is a dynamic process that changes over time and is subject to strong bias, with white coat effects that typically increase adherence in the 24-48 hours before a scheduled visit to the clinic or laboratory [11-13]. The electronic bottle cap, such as the medication event monitoring system device, has also been used as a potential surrogate for drug dosing [14]. However, several limitations have been reported with this methodology, including mismatches between electronic cap opening and actual intake (more than one dose taken or no dose taken) and patients obviating electronic assessment by decanting pills from a bottle having an electronic cap to an alternative daily decanter or a weekly pill box [4-6].

Even fully implemented DOT has drawbacks for adherence. DOT by definition does not allow naturalistic observation of an individual patient's drug taking behaviors. One concept emphasized in medication adherence literature is the link between a patient's sense of self-efficacy, that is, an individual's perception of their ability to perform a specified behavior or set of behaviors [15], and their ability to execute satisfactory adherence. In one recent study comparing modified DOT with self-administered drugs, Gross et al. observed that DOT does not enable a patient to learn to manage their own drug taking behavior, a factor that may detract from improvements in medication compliance in the long term [16].

There is clearly an unmet need for an alternative to DOT that can reliably and efficiently confirm medication ingestion events and provide verification of TB treatment adherence and course completion. This tool should be accurate and easy for both the patient and healthcare worker to use, and ideally should provide feedback to the patient to guide their compliance with TB treatment.

2.2 Rationale

A digital health feedback system (DHFS) (Proteus Digital Health, Inc., Redwood City, CA, USA) has been developed to provide wirelessly observed therapy (WOT) as a reliable, efficient, scalable, and cost-effective alternative to DOT for sharing complete and accurate medication data in near real-time among patients, prescribing physicians, public health workers, pharmacists, and other healthcare providers. WOT represents a potential new paradigm for TB therapy monitoring, which can be utilized by public health workers and outreach workers to

confirm TB treatment. **The ultimate goal of this protocol is to utilize WOT as an alternative to DOT for daily real-time assessment of TB medication adherence in an ambulatory setting, providing individualized mobile healthcare using prompt, targeted feedback for the management of TB infected patients.** This pilot trial provides an opportunity to lead the way forward in the clinical practice of TB control with this innovative U.S.-developed technology. The results from this trial will demonstrate the feasibility and the utility of this technology to other countries that have a higher TB burden.

DHFS represents the first technology that allows date- and time-stamping of actual ingestions of oral medications, instead of surrogate measures of ingestion [17]. It can also provide specific information regarding the types and doses of multiple drugs taken simultaneously. DHFS allows secure medication-taking data to flow to patients, prescribing physicians, pharmacists, and other healthcare providers [17, 18]. The data shared between patients and healthcare providers affords the opportunity 1) to assess the regularity of medication-taking and 2) to support patient medication taking behavior in near real time to encourage optimal adherence.

DHFS consists of two main components: 1) an ingestion sensor, and 2) a wearable sensor to detect and record ingestion and physiological activity. The ingestion sensor is smaller than a sesame seed (1.0 mm x 1.0 mm x 0.45 mm) and composed of minerals and metals in the human food chain. The sensor can be encapsulated with prescribed medication. When ingested, the sensor utilizes gastric fluid and electrolytes to communicate information to an adhesive-backed wearable sensor. The wearable sensor is worn on the torso and confirms and records the identity, date, and time of sensor ingestion. The wearable sensor interprets each individual ingestion sensor's information as unique. The communication process lasts approximately 5-7 minutes and is unnoticed by, and not detectable beyond, the system user. Like an ingested sesame seed, the remainder of the ingestion sensor is then passed and excreted with the stool.

In this manner, DHFS is capable of directly confirming when, how much, and how many doses of prescribed medication have been ingested. The wearable sensor decodes and records the information from the ingestion sensor, and date- and time-stamps ingestions and physiological metrics such as rest and activity. All of the recorded data is sent wirelessly using encryption to a computing device (such as a mobile device or a personal computer) and is subsequently uploaded to a secure, centralized data storage location. The data can then be formatted and directed back in a similarly secure manner to the patient or those designated by the patient, so they can keep track of their own medication-taking and other activities of daily living [17-19].

The DHFS has been studied in healthy volunteers, as well as in patients with TB, heart failure, hypertension, diabetes, schizophrenia, and bipolar disorder as their primary disease. The ingestion sensor has been cleared via the 510(k) pathway and has received the CE mark in the EU. The following is an excerpt from the FDA-cleared device label which summarizes the technical performance: "A total of 412 study subjects have participated in Pill ingestion studies representing 20,993 ingestible sensor ingestions. In comparison with direct observation, the ingestible sensor was detected in 97.3% of ingestions, with correct identification in 100%." The latest configuration of the system, which includes wearable sensor version RP4 and the Miniature Ingestible Event Marker Tablet dose form, underwent post-approval, formative testing, in which sensitivity for ingestion sensor detection versus directly observed ingestion was 99.4% (95% CI 97.9-99.8%), and there were no false positive detections. Regarding ingestion

sensor safety, the following is also abstracted from the FDA-cleared device label: "nausea/vomiting occurred in 1%, constipation in 0.5%, asthma attack in 0.2%, abdominal cramping in 0.2%, non-cardiac chest pain in 0.2% and bitter taste in mouth in 0.2%. None of these adverse events were considered serious and all resolved spontaneously" (Ingestible Sensor Investigator's Brochure, Rev 4).

Wearable sensor version RP2 and RP3 have been cleared via the 510(k) pathway and have received the CE mark in the EU. The following is an excerpt from the FDA-cleared device label, which summarizes the safety of these earlier wearable sensor versions: "A total of 492 study subjects have participated in clinical studies representing 6,407 days of Patch (wearable sensor) use with or without ingestible sensor ingestion. Sixty-two (12.6%) of these users experienced adverse events related or possibly related to the Patch. Sixty-one of these AE were self-limited skin rashes localized to the Patch placement site, and 1 was a self-limited episode of pain at the Patch location unassociated with skin rash. None of these adverse events were considered serious." The latest wearable sensor version (RP4), which remains investigational at present, has undergone testing in 38 subjects. Four AE were reported (10.5% of subjects), all localized to the wearable sensor placement site and mild in severity. Three of these AE were erythematous rashes at the wearable sensor location; one of these rashes transiently manifested itself as non-erythematous skin discoloration ("secondary NESD") on the path to full resolution. The fourth AE was an episode of "primary NESD" (i.e., there was no antecedent erythematous rash reported at the wearable sensor location). Primary and secondary NESD have been encountered with previous wearable sensor versions. The investigator hypothesized that per-protocol premature removal of the wearable sensor in these studies (typically after several hours, as opposed to intended 7-day wear) significantly contributed to the occurrence of these four RP4-related AE (Wearable Sensor Investigator's Brochure, Rev 4).

A proof of feasibility study has been conducted in 30 TB patients and published from two clinical sites in the U.S., using an earlier prototype of the current WOT system. This was a prospective, non-randomized, single-blinded study to evaluate the feasibility and safety of an early version of the DHFS in patients with active TB and in the continuation phase of treatment. Study participants were monitored during 10 consecutive daily DOT visits where ingestible event markers (IEM) were ingested separately at the time of medication dosing. At each visit, participants ingested 2 tablets along with their TB medications. Of the 20 tablets ingested by each patient (over 10 visits), 18 had IEM attached and 2 placebo tablets contained no IEM. The IEM system showed high positive and negative detection accuracies in identifying medication ingestions [20]. The DHFS has been updated substantially since this early study.

This pilot trial project will utilize DHFS to assess medication adherence to ingestion sensor-enabled Rifamate (IS-RM) reliably, in an ambulatory setting in order to accurately characterize medication taking behavior of adult patients undergoing treatment for TB that includes at least INH and RIF. The system will provide a means of directly confirming medication adherence and identifying non-adherent patients to County healthcare workers, who can then provide prompt adherence intervention, thereby preventing treatment failure. The system can also provide individualized healthcare using prompt-targeted feedback tailored to individual TB patients.

The ingestible sensor will be over-encapsulated with a fixed-dose combination of isoniazid-rifampin (Rifamate), IS-RM; thus DHFS will capture actual ingestion of IS-RM. In this

feasibility study, subjects meeting eligibility criteria will initially have ingestion of IS-RM confirmed by DHFS for two weeks with simultaneous healthcare worker witness of actual IS-RM medication ingestion to evaluate the positive detection accuracy of DHFS. Alternatively, during Phase I, subjects may take Rifamate with co-ingestion of an encapsulated edible sensor. Subsequently, those eligible subjects who wish to continue will be randomized to either DHFS or SOC DOT. DHFS has previously been studied for up to 4 months in patients with chronic disease. Phase 2 of this trial will look at persistence of use from 4 weeks until completion of TB treatment (up to a maximum of 12 months on study).

Per CDC guidelines, DOT can take place five, three, or two times per week, with the medication dose adjusted to the frequency of medication taking. Although DOT is the gold standard for monitoring therapy, it can be difficult to execute perfectly, since subjects may miss DOT appointments for vacation, work, or other reasons, and conversely DOT workers may miss appointments due to overscheduling or transportation failure. Thus, the adherence metrics chosen for this study need to carefully take into account the unique nature of DOT, as adherence determined by the number of witnessed doses ingested. The optimal adherence metric for this study is the total number of actual witnessed dose ingestions divided by the total number of prescribed witnessed dose ingestions, captured by DOT or via DHFS. This parameter, the percentage of witnessed doses, will be reported for the duration of the study (per week, per month and over the entire study period). The percentage of witnessed doses in this trial captures effective monitoring of TB treatment via DOT and DHFS, and allows calculation of adherence with SOC DOT at varying intervals, e.g., five, three, or two times per week.

TB treatment has largely grown up through convention and analysis of treatment failures in traditional clinical practice, rather than extensive clinical trials with pharmacokinetic and therapeutic drug monitoring. Thus patterns of drug administration given via DOT five, three, or two times per week have been deemed acceptable based on treatment failure rates, with adherence $\geq$ 80% of prescribed dosing estimated by key opinion leaders as adequate for TB treatment [21]. Daily DOT may give more favorable pharmacokinetic profiles for isoniazid and rifampin with their relatively short half-lives (isoniazid-1.5 hours [fast acetylators] to 4 hours [slow acetylators]; rifampin-3 hours). Random and sparse sampling of therapeutic drug concentrations may confirm adherence monitoring data obtained via DHFS and SOC DOT; and pharmacokinetic evaluation of daily and five, three, and two times weekly dose administration may be analyzed. Stored plasma samples in this trial may allow future pharmacokinetic analysis with random sampled dosing history data providing the foundation for sound pharmacological interpretation of dose- or exposure-dependent variations in drug response in ambulatory care of TB.

3.0 STUDY DESIGN

This study is a prospective, two phase, open-label feasibility study with lead in (Phase 1) and randomized (Phase 2) components to evaluate the utility of the DHFS for monitoring TB treatment. In Phase 1, all subjects will receive DHFS IS-RM (or Rifamate with co-ingestion of an encapsulated edible sensor) and a series of doses will be witnessed in order to determine the detection accuracy of the system, as well as to evaluate subject acceptance and ability to use the system. In Phase 2, those randomized to intervention will continue to use the DHFS IS-RM and control subjects will receive SOC DOT.

3.1 Non-Significant Risk Study

To date, all of the clinical investigations in the U.S. of the DHFS and its wearable sensor component have been designated as non-significant risk studies, as they have met the established regulatory criteria for a non-significant risk device study. Specifically, the system and the wearable sensor component are not:

- an implant used to support or to sustain human life
- being used for substantially diagnosing, curing, mitigating or treating disease, or preventing impairment of human health, or
- a potential serious risk to the health, safety or welfare of subjects.

No serious adverse events (SAE) and no unanticipated adverse device effects (UADE) have been reported. The vast majority of the non-serious, device-related AE that have been reported in completed studies have been categorized as mild in severity. Please see Investigator's Brochure for additional details.

3.2 Study Population

Study participants will be approximately 100 adult patients being treated for TB that includes at least isoniazid and rifampin, or Rifamate and who have at least 6 weeks remaining of treatment.

Eligible subjects will be at least 18 years of age, have sputum smear negative or culture negative TB, have no evidence of drug resistant TB, and have complete blood count (CBC) and comprehensive panel (CMP) values that fall within parameters specified in Section 4.1 (Inclusion Criteria). In cases where abnormalities in these laboratories exist, the study Principal Investigator or co-investigator physicians must be contacted to directly determine whether the observed lab abnormalities exclude patient from enrollment. Females of child bearing age must be willing to use contraception during the study. Subjects must be able to understand written and verbal information regarding DHFS use, be able to use a mobile device and wear a patch sensor, and have the ability to give written informed consent.

Criteria for exclusion will include pregnancy, or being fertile and not using a clinically accepted means of birth control for the duration of the study, use of prohibited medications (see Sections 4.2 and 5.4.2), known hypersensitivity to skin adhesives, and inability to understand instructions regarding and use of the components of the DHFS.

All eligible subjects willing to participate in the study will sign an informed consent. Following consent and pre-study interview, subjects will participate in a Phase 1, two-week investigation of the positive detection accuracy and characteristics of use of DHFS in the TB patient population, using DHFS IS-RM (or Rifamate with co-ingestion of an encapsulated edible sensor) with simultaneous DOT. Patients deemed eligible after Phase 1 and who elect to continue will then be randomized to DHFS IS-RM or SOC DOT delivered by County healthcare workers.

The study will be conducted at the UCSD AVRC, at PHS, and OCHCA. Subjects will be recruited from County TB clinics in San Diego and Orange Counties, San Ysidro Health Centers, and UCSD. Study enrollment will take place from the period of 09/01/2013 through 12/01/2015.

Subjects will be enrolled into the study for a minimum of 6 weeks, and will end the study at completion of TB treatment (up to a maximum of 12 months on study).

3.3 Recruitment

The study will be advertised in flyers placed at PHS, OCHCA, San Ysidro Health Centers, and at UCSD. County outreach workers and healthcare personnel will inform all of their patients of this study, as will San Ysidro Health Centers and UCSD personnel involved in TB care. Interested patients will be directed to contact AVRC study personnel by phone. When patients interested in participating call, AVRC personnel will describe the study. They will inform patients that they may enroll in the study to undergo a two-week investigation of use and accuracy of DHFS IS-RM and then if willing and eligible, be randomized to the intervention with remote WOT via DHFS IS-RM, or to SOC DOT. The AVRC will establish the eligibility of potential participants (see above).

If a patient wants to participate, the study coordinator will then conference with the public health nurse and DOT outreach workers to review the patient's suitability for the study, including adequacy of living situation and ability to use mobile technology. If the patient is willing to participate and is recommended by PHS as able to participate, the patient will be scheduled for an appointment with AVRC personnel. At this appointment, AVRC personnel will again describe the study to the patient and confirm eligibility criteria (see above). Screening will include documentation of TB, smear negative or culture negative status, and resistance status as recorded, clinical assessment with targeted exam, concomitant medications and toxicities, and laboratory values obtained from CLIA certified clinical laboratories, within the last 30 days, or by County or AVRC laboratory if routine labs are unavailable (only lab values drawn by OCHCA staff will be used for Orange County participants), including complete blood count (CBC) with differential, chemistry panel and liver function tests (CMP), (abnormalities in these laboratory values will be reviewed by the PI or physician co-investigators prior to enrollment), and pregnancy test (as appropriate). The interviewer will assess the subject's attitude to mobile devices, understanding and experience of TB medications thus far, and whether they would be able to understand DHFS instructions of use and be willing to use all the components of the system. If the patient is viewed as able and eligible, the AVRC personnel will supervise signature of informed consent.

Following informed consent, subjects will be assigned a participant identification number. Data will be collected on demographics (age, race/ethnicity, gender, employment, education level), co-morbidities, and concurrently administered medications, and will include review of information from County records and medical records. Each subject will then participate in a two-week investigation of the positive detection accuracy and characteristics of use of DHFS in the TB patient population (Phase 1). Following the Phase 1 lead in, subjects who remain eligible and who elect to continue will then be randomized in a 2:1 ratio to either an intervention group with DHFS IS-RM or a control group receiving SOC DOT with isoniazid plus rifampin, or Rifamate. Subjects will be accrued until 50 subjects are in the intervention and 25 subjects are in the control arm.

3.4 Study Conduct and Data Collection

Prior to the commencement of the study, AVRC personnel and County healthcare providers will be trained in set up and use of the PDH DHFS medication monitoring system. This includes information on the ingestion sensor, the wearable sensor, use of study computerized devices, and study drug. Healthcare providers will also be given guided instruction on the provider data view, which will display daily patch adhesion, patch sensing, and ingestion data for each study subject using DHFS. The study coordinator and County healthcare workers will be shown how to check the PDH website containing daily medication ingestion and wearable sensor data. The study coordinator will be transcribing this information manually into the study database. All data will be entered by study staff under the subject's participant number. Study data collection is described below in Section 10. County healthcare workers will be entering daily medication ingestion confirmation information into the County health records as is current practice (see below). Whenever possible, data will be collected from County records and medical records, for the duration of the subject's participation in the study.

Following subject consent, the study will proceed as described below.

3.5 Study Design

Phase 1: A two-week period where the subjects receive DHFS IS-RM (or Rifamate with co-ingestion of an encapsulated edible sensor), in conjunction with witnessed medication doses to determine the positive detection accuracy and characteristics of use of DHFS in the TB patient population.

Phase 2: A period of TB treatment monitoring randomized to DHFS IS-RM or SOC DOT, for a minimum of 4 weeks and continuing until completion of TB treatment (up to a maximum of 12 months on study).

These study periods are designed to capture the following information. Phase 1 will gather information on the verification of DHFS ability to capture medication ingestion events alongside simultaneous witnessed ingestions. This Phase incorporates DHFS use at the AVRC or OCHCA clinic with simultaneous witnessed ingestions by clinic staff to ascertain the positive detection accuracy (with calculation of sensitivity and specificity), system function, and the subject's ability to interface with the DHFS system as currently designed. Phase 2 represents a period of persistence with DHFS IS-RM or SOC DOT and monitoring of patient medication taking behavior until the completion of TB consolidation treatment or termination on the study.

In Phase 1 (Weeks 0-2), all subjects will use DHFS and receive the study drug in conjunction with DOT. The wearable sensor will be changed weekly (or earlier if needed) by the subject themselves under the supervision of study personnel. Subjects will be required to visit the AVRC two times a week during this phase to complete DHFS/DOT doses. In the case of Orange County participants, AVRC personnel will conduct all study visits at the OCHCA clinic. County outreach workers will also provide at least three visits per week during Phase 1, providing a combined minimum of five days of DOT per week in conjunction with study drug ingestion. Upon completion of four successful ingestions of Rifamate with concurrent use of DHFS and witnessed DOT during study visits, and two successful patch changes performed under

observation by study personnel, Phase 1 of the study will be completed. The first patch change may include minimal coaching, but no intervention by study personnel. The second patch change must occur without any coaching or intervention by study personnel. Subjects deemed eligible by study personnel and who wish to continue will be randomized and proceed to Phase 2.

On the last day of Phase 1, the subject will take DHFS IS-RM (or Rifamate with co-ingestion of an encapsulated edible sensor). On this same day, the investigator will 1) access the Core Medical Device (CMD) viewer that is available on the subject's mobile study device, and 2) obtain the dates and times from the CMD of all Rifamate ingestions that were detected by the DHFS, with the exception of the final day of Phase 1. The dates and times of all DHFS detections that were logged on the CMD during Phase 1 will be entered into the study database for subsequent PDA analysis.

In Phase 2, subjects are randomized 2:1 to the Intervention Group that will use DHFS with IS-RM without concurrent DOT, or to the Control Group that will receive standard isoniazid plus rifampin, or standard Rifamate, per SOC that includes DOT from County healthcare workers.

3.5.1 Intervention Group

In intervention subjects, the patch monitor will be changed weekly by the subject themselves or under the supervision of the AVRC/County healthcare worker if requested. Subjects will be contacted weekly for review of side effects and any questions by AVRC and will be able to contact the AVRC and County healthcare worker at any time with questions regarding the intervention. Subjects will continued to be followed by County healthcare per their usual routine (i.e., County nurse visit once a week, physician visit once a month, and County outreach worker visits as directed by County nurse). All phone calls between the subject and the AVRC or County healthcare workers including medical information, such as physical symptoms possibly associated with side effects or other medical complaints, will be recorded as per the Schedule of Events (see Section 6) and communicated to PHS, OCHCA, or the physician with primary responsibility for the subject's TB treatment. All phone calls between the subject and the AVRC or County healthcare workers related to the function of the DHFS, will be directed to and dealt with by the AVRC in conjunction with PDH personnel.

The AVRC study coordinator and County healthcare worker will review the subject ingestion log on the PDH website daily on business days to record the subject's wirelessly confirmed dosing. This follows current County practices. The wirelessly confirmed dose will be entered by AVRC study staff into the study database via a secure user-authenticated website designed and hosted by the UCSD Center for AIDS Research Bioinformatics and Information Technologies (BIT) Core. The identity of staff recording the wirelessly confirmed dose will be recorded in the study database as the user who logged into the secure website to enter the data. The County healthcare worker will enter the subject's wirelessly confirmed dosing into the County medical records daily, following current County practices. Each site should check daily to see that the subject's data has

been recorded properly into the study database (by the AVRC) and medical record (by PHS or OCHCA).

In Phase 2, if ingestion of DHFS IS-RM cannot be wirelessly confirmed on any business day, the subject will be contacted within 24 hours. During this call, information will be obtained to clarify whether there is a malfunction of the DHFS or non-adherence to medication has occurred. In case of malfunction of the DHFS, the AVRC in conjunction with PDH will address the problem. In the case of medication non-adherence in the absence of DHFS malfunction, such as due to medication side effects, County healthcare workers will ascertain the reasons for non-adherence and follow County protocol. This process is summarized in Figure 2 below.

Figure 2. AVRC/County Outreach Worker Algorithm Used for Wirelessly Confirmed Ingestions

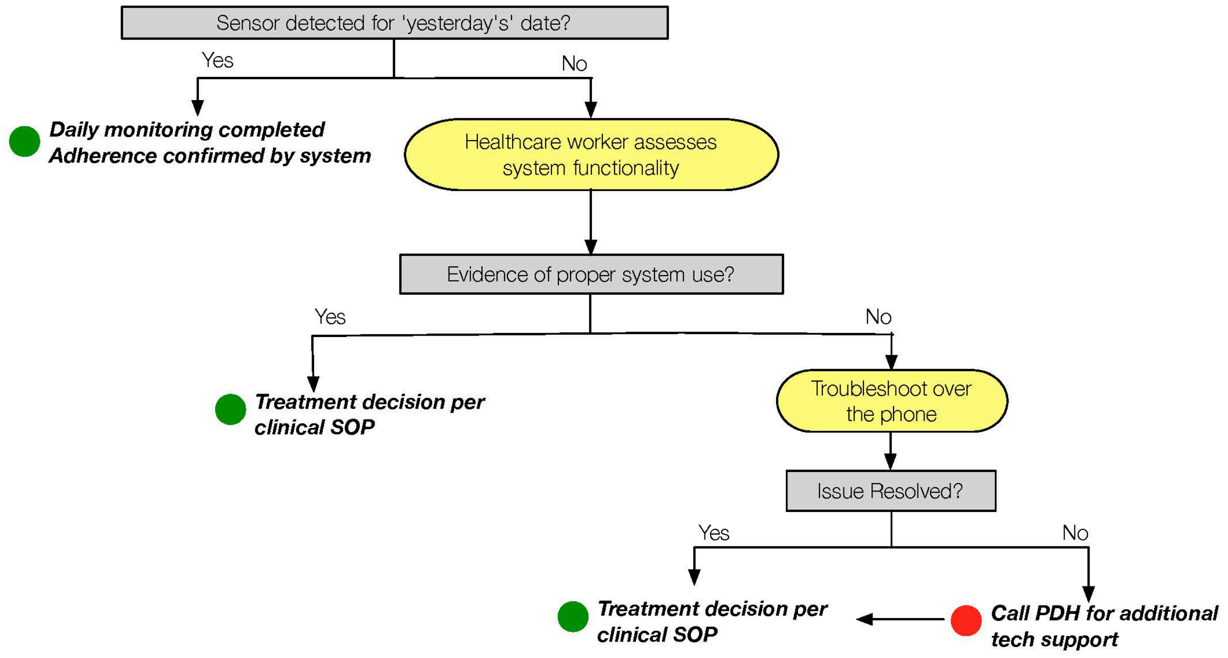

Safety laboratory values will be reviewed at Weeks 2, 4, 8, and every 4 weeks until the completion of TB treatment (up to a maximum of 12 months on study) and at the end of study, or at frequency of PHS or OCHCA SOC. For the full schedule of events at these weeks, see Section 6.1.

In Phase 2 on Day 0, Week 8, and at the end of study, subjects will be asked to complete the following questionnaires: habitual self-control, self-efficacy beliefs, depression scale (PHQ-9), alcohol use (AUDIT), and drug use (DAST-10). Questionnaires will be available in English and Spanish; for subjects with other

first languages, questionnaires will be administered with the aid of an interpreter, if necessary. All self-report questionnaires will be completed via a study tablet device. Questionnaires will automatically upload to a secure server after completion.

As indicated above, each intervention subject will also be followed by County healthcare workers, who will collect and follow data on intervention subjects following County routine practice, including data from medical records, adherence data as witnessed ingestions, cost data, any toxicity data and side effect data pertaining to non-DHFS medical treatment, including number of clinic visits and number of hospitalizations related to TB treatment, per patient during the study period. AVRC study staff must ensure that data from County records and medical records are on study CRFs and entered into the study database.

3.5.2 Control Group

Subjects randomized to the control arm will receive SOC DOT with isoniazid plus rifampin, or Rifamate via the County healthcare workers. Safety laboratory values will be reviewed as per County SOC. At Phase 2 Day 0, Week 8, and at subject's termination on the study, the following questionnaires will be administered: habitual self-control, self-efficacy beliefs, depression scale (PHQ-9), alcohol use (AUDIT), and drug use (DAST-10). Questionnaires will be available in English and Spanish; for subjects with other first languages, questionnaires will be administered with the aid of an interpreter, if necessary. These data will be identified by subject's participant number only and will not be associated with the subject's name. All self-report questionnaires will be completed via a study tablet device. Questionnaires will automatically upload to a secure server after completion.

County healthcare workers will collect and follow data on control subjects following County routine practice, including data from medical records, adherence data as prescribed DOT (two, three, or five times per week), actual witnessed ingestions, cost data, any toxicity data and side effect data pertaining to treatment, including number of clinic visits and number of hospitalizations related to TB treatment, per patient during the study period. AVRC study staff must ensure that data from County records and medical records are on study CRFs and entered into the study database.

3.5.3 Study Medication

In Phase 1, oral Rifamate will be supplied by the AVRC pharmacy. All medications will be over-encapsulated by the AVRC pharmacist. PDH will supply the ingestible sensor and the AVRC pharmacist will over-encapsulate Rifamate with the ingestible sensor (or in Phase I, the ingestible sensor alone) using an appropriate size capsule, manufactured by Capsugel Inc., according to Standard Operating Procedures. Thus the AVRC will dispense over-encapsulated IS-RM or

Rifamate with co-ingestion of an encapsulated edible sensor to all Phase 1 subjects.

In Phase 2, subjects in the intervention arm will be dispensed study drug, IS-RM, following the process described above, by the AVRC pharmacy to coincide with AVRC visits or study visits with AVRC personnel in Orange County. Control subjects will have medications per SOC prescribed by PHS or OCHCA and dispensed by a public health pharmacy for two, three, or five times a week DOT.

3.6 Study Outcome Data

Detection Rates: Detection rates of ingested study drug, sensitivity and specificity calculation using data derived from Phase 1 of study.

Positive Detection Accuracy (PDA): PDA is defined as DHFS detection of the ingestion of a dose of IS-RM when compared to a witnessed ingestion of the same dose of medication. For purposes of PDA analysis, a dose is as a single IS-RM capsule. A minimum of 280 dose ingestions are required to generate sensitivity and specificity of the System. The expected treatment for study subjects is two IS-RM capsules daily.

Persistence of WOT signal transfer and patch function over period of study providing confirmed ingestion of TB medication will be reported as the percentage of witnessed doses captured. For the DHFS, the percentage of witnessed doses by DHFS is defined as the total number of dose ingestions confirmed by DHFS, divided by the total number of prescribed witnessed dose ingestions for TB treatment. For SOC DOT, the percentage of witnessed doses is defined as the total number of dose ingestions that are actually witnessed by a healthcare worker, divided by the total number of prescribed witnessed dose ingestions for TB treatment. This endpoint will be calculated from baseline to Weeks 4, 8 and end of follow-up.

For additional details, see Primary Endpoints in Section 9.2.1, and Secondary Endpoints in Section 9.2.2.

3.7 Discontinuation for Medication Related Toxicity

If the subject develops a toxicity that results in discontinuation of isoniazid and rifampin, or Rifamate, then an alternative medication regimen will be found and started as soon as feasible. The medication will not be supplied by the study. These patients will be required to permanently discontinue their study regimens but will continue to be followed for the duration of the study.

3.8 Plasma Storage

Patients seen at the AVRC (this excludes subjects drawn from OCHCA) will undergo random collection of plasma, which will be stored to later run drug levels of rifampin. The time and date of the three previous doses of TB medications should be recorded.

4.0 SELECTION AND ENROLLMENT OF SUBJECTS

4.1 Inclusion Criteria

4.1.1 Basic competency in understanding written and verbal information as it applies to DHFS use.

4.1.2 Persons undergoing treatment for TB that includes at least isoniazid and rifampin at the time of entry to Phase 1; of note, patients must be sputum smear negative or culture negative at the time of study entry.

4.1.3 Laboratory values obtained by screening laboratories within 30 days of entry:
- Absolute neutrophil count (ANC) ≥ 1,000/mm^3.
- Hemoglobin ≥ 9.0 g/dL.
- Platelet count ≥ 75,000/mm^3.
- AST (SGOT), ALT (SGPT), and alkaline phosphatase ≤ 3 x ULN.
- Total bilirubin ≤ 1.5 x ULN and direct bilirubin.

4.1.5 Females of childbearing potential must agree to use contraception throughout the study period.

4.1.6 Men and women age ≥ 18 years.

4.1.7 Eligible for anti-mycobacterial medications and in possession of prescriptions for isoniazid and rifampin, or Rifamate, as appropriate.

4.1.8 Willing to follow all protocol requirements.

4.1.9 Ability to use mobile device per investigator determination, and to wear PDH wearable sensor (i.e., no skin conditions precluding use).

4.1.10 Ability and willingness of subjects to give written informed consent.

4.2 Exclusion Criteria

4.2.1 Female who is pregnant or breast-feeding, or of childbearing potential and has a tuberculin positive test at screening and disagrees to use contraception throughout the study period.

4.2.2 Use of any of the prohibited medications or other non-informed medications (Section 5.4.2) within 30 days of study entry.

4.2.3 Known hypersensitivity to any of the study drugs.

4.2.4 Known sensitivity to skin adhesives.

4.2.5 Serious illness requiring systemic treatment and/or hospitalization until subject either completes therapy or is clinically stable on therapy, in the opinion of the investigator, for at least 30 days prior to study entry (Day 0).

4.2.6 Evidence of any anti-mycobacterial resistance, clinical or genetic, prior to study entry. Resistance testing results must be available for review by the site investigator and study protocol team prior to enrollment to ensure that no exclusionary resistance exists.

4.2.7 Active drug or alcohol use, or dependence, or other conditions that, in the opinion of the site investigator, would interfere with adherence to study requirements.

4.3 Study Enrollment Procedures

4.3.1 Prior to implementation of this protocol, sites must have the protocol and consent form approved by their local institutional review board (IRB). Once a candidate for study entry has been identified, details will be carefully discussed with the subject. The subject will be asked to read and sign the consent form that was approved by the local IRB.

4.3.2 A patient identification number (PID) will be assigned to each patient screened for the study. PIDs should not be reassigned even if the patient fails to enter the study. The PID must be included on every CRF and patient blood sample. BIT Core must maintain a master list of PIDs in a central location. The patient registration and inclusion/exclusion CRF must be completed on the online system (cfar.ucsd.edu/intranet).

5.0 STUDY TREATMENT

5.1 The Ingestible Sensor System

The system consists of 1.0 mm x 1.0 mm x 0.45 mm ingestible sensor and an on-body wearable sensor. The ingestible sensors are activated by gastric fluids, independent of the acidity level, and communicate unique identifying signatures to the body surface. The system uses a conductive method of communication, not radio frequency, which ensures the information is confined to the body of the user and preserves privacy. The wearable sensor counts the number of times each unique signature is received. For pre-study, the ingestible sensors were attached to inert tablets and co-ingested with the TB medications. The system recorded the date and time of an ingestion event after a unique signature was received 10 times. The ingestible sensors are designed to communicate for approximately 7 minutes after which they are inactive and get eliminated in the feces. The system is capable of identifying and differentiating among multiple simultaneously ingested sensors.

5.2 Regimens, Administration, and Duration

Study Treatment is defined as:

DHFS Group: over-encapsulated ingestion sensor plus a fixed-dose combination of isoniazid-rifampin (Rifamate) (study supplied) (alternatively in Phase 1, Rifamate with co-ingestion of an encapsulated edible sensor)

SOC Control Group: isoniazid plus rifampin, or Rifamate (by prescription)

5.2.1 Regimens

DHFS Group:
Rifamate (combination of isoniazid 150 mg and rifampin 300 mg) over-encapsulated with ingestion sensor - 2 capsules orally QD (provided by the study through the AVRC pharmacy)

SOC Control Group:
isoniazid 300 mg -1 tablet orally QD plus rifampin 300 mg - 2 capsules orally QD, or Rifamate (combination of isoniazid 150 mg and rifampin 300 mg) - 2 capsules orally QD (provided by the PHS, OCHCA, or study physician)

5.2.2 Administration

Isoniazid and rifampin, or Rifamate, will be administered orally as described above, preferably on an empty stomach (at least one hour prior to or two hours after a meal) first thing in the morning.

5.2.3 Duration

Subjects will receive study treatment for a minimum of 6 weeks up to a maximum of 12 months on study, depending on time left to complete treatment.

5.3 Study Treatment (DHFS IS-RM) Formulation and Preparation

5.3.1 Formulation

Isoniazid-rifampin (Rifamate) fixed dose formulation capsules over-encapsulated with ingestible PDH sensor. Store at 25^0C (77^0F), excursion permitted to 15-30^0C (59-86^0F) (see USP Controlled Room Temperature); protect from excessive heat and humidity.

5.3.2 Preparation

Ingestible PDH sensor and isoniazid-rifampin (Rifamate) will be over-encapsulated with Capsugel gelatin capsules, and dispensed by the AVRC pharmacy. The final study product will be packaged in white, high density polyethylene bottles. Each bottle will contain a sufficient quantity of study drug to last until the subject's next scheduled visit.

5.4 Study Product Supply, Distribution, and Accountability

5.4.1 Study Product Supply/Distribution

For the DHFS group, ingestion sensors will be purchased from Proteus Digital Health, Inc. and anti-TB drugs (Rifamate) and over-encapsulation will be provided by the AVRC pharmacy. The study product (IS-RM) will be supplied by the study.

For the control group receiving SOC, isoniazid and rifampin, or Rifamate, will not be supplied by the study, but will be made available to subjects via prescription at a public health pharmacy.

5.4.2 Study Product Accountability

The clinical site pharmacist is required to maintain complete records of all study products received, compounded (over-encapsulated), and subsequently dispensed for this study. All unused study products must be returned to the sponsors after the study is completed or terminated.

5.5 Concomitant Medications

5.5.1 Required Medications

WHILE PARTICIPANTS ARE TAKING ISONIAZID OR RIFAMATE (isoniazid in combination with rifampin), PYRIDOXINE (VITAMIN B6) 25 MG OR 50 MG MUST ALSO BE GIVEN ONCE DAILY.

5.5.2 Prohibited Medications:

- All investigational drugs.
- Any immunomodulators.
- Systemic cytotoxic chemotherapy.
- The following antiviral drugs: ritonavir, Stribild®, atazanavir, darunavir, fosamprevavir, saquinavir, tipranavir, delavirdine, etravirine, and rilpivirine.
- All herbal products should be avoided because of the unknown drug interactions between herbal products and TB drugs used in this study.

5.5.3 Precautionary Medications

5.5.3.1 Precautionary Medications with Rifampin

Medication Class	Precautionary Medications
ACE Inhibitors	Enalapril
Antiarrhythmics	Disopyramide<br>Mexiletine

Medication Class	Precautionary Medications
	Quinidine<br>Tocainide
Antibiotics	Chloramphenicol<br>Clarithromycin<br>Dapsone<br>Doxycycline<br>Fluoroquinolones
Anticoagulants	Warfarin
Anticonvulsants	Phenytoin
Antimalarials	Quinine<br>Atovaquone
Antiretrovirals	Efavirenz<br>Nevirapine<br>Raltegravir<br>Zidovudine<br>Dolutegravir
Antipsychotics	Haloperidol
Azole Antifungals	Fluconazole<br>Itraconazole<br>Ketoconazole
Barbiturates	Phenobarbital
Benzodiazepines	Diazepam
Beta-Blockers	Propranolol
Calcium Channel Blockers	Diltiazem<br>Nifedipine<br>Verapamil
Cardiac Glycoside Preparations	Digoxin
Corticosteroids	Prednisone
Fibrates	Clofibrate
Hormonal Contraceptives/Progestins	Ethinyl estradiol<br>Levonorgestrel
Oral hypoglycemic agents	Sulfonylureas
Immunosuppressants	Cyclosporine<br>Tacrolimus
Methylxanthines	Theophylline
Narcotic analgesics	Methadone
Phosphodiesterase-5 (PDE-5) Inhibitors	Sildenafil
Thyroid preparations	Levothyroxine

Medication Class	Precautionary Medications
Tricyclic Antidepressants	Amitriptyline<br>Nortriptyline

5.5.3.2 Precautionary Medications with Isoniazid:

- Carbamazepine
- Chlorzoxazone
- Disulfiram
- Ketoconazole
- Phenytoin

The physician responsible for prescribing TB medications to subjects should be made aware of the following: It may be necessary to adjust the dosages of some medications when given concurrently with isoniazid/rifampin. To avoid adverse drug interactions, package inserts of anti-TB agents and other concomitant medications should be referenced whenever a concomitant medication is initiated or dose changed, to avoid drug interaction AEs.

5.5.4 Food Interactions

Prescribing physicians should be aware that since isoniazid has some monoamine oxidase inhibiting activity, an interaction with tyramine-containing foods (cheese, red wine) may occur. Diamine oxidase may also be inhibited, causing exaggerated response (e.g., headache, sweating, palpitations, flushing, hypotension) to foods containing histamine (e.g., tuna, other tropical fish). Tyramine- and histamine-containing foods should be avoided.

6.0 CLINICAL AND LABORATORY EVALUATIONS

6.1 Schedule of Events

Table 1.

Evaluation	Screening	Phase 1				Randomization	Phase 2							Completion of Treatment/ End of Study or Premature Discontinuation
		Week 1 Day 0	Week 1 Visit 1	Week 2 Visit 2	Week 2 Visit 3		Day 0	Daily	Weekly	Wk 2	Wk 4	Wk 8	Wk 12 and every 4 weeks	
Visit Window		Within 45 days of Screening								± 7 days	± 7 days	± 14 days	± 14 days	
Informed Consent	X													
Demographics and Language	X													
Documentation of TB	X													
Medical History	X													
Medication History (last 30 days)	X													
Toxicity/AE Evaluation	X	X	X	X	X		X			X	X	X	X	X
Targeted Exam	X	X					X			X	X	X	X	X
Concomitant Meds (since last visit)		X	X	X	X		X			X	X	X	X	X
Initiation of study medication		X					X*							
Randomization						X								
**PDH System**														
Subject training		X	X				X*							
Observation with PDH Patch		X	X	X	X		X*			X*	X*	X*	X*	X*
Patch change (by subject)		X		X			X*		X*					
Witnessed Rifamate doses		X	X	X	X		X*							
Adherence Intervention Based on PDH Sensor**								X*						

Evaluation	Screening	Phase 1				Randomization	Phase 2							Completion of Treatment/ End of Study or Premature Discontinuation
		Week 1 Day 0	Week 1 Visit 1	Week 2 Visit 2	Week 2 Visit 3		Day 0	Daily	Weekly	Wk 2	Wk 4	Wk 8	Wk 12 and every 4 weeks	
**Laboratory Evaluations**														
Hematology§	X						X			X	X	X	X	X
Liver Function Tests§	X						X			X	X	X	X	X
Chemistry§	X						X			X	X	X	X	X
Pregnancy Testing (and whenever pregnancy is suspected)	X	X												
Stored Plasma/Serum λ					X		X				X	X	X	X
**Assessments**														
Adherence Self-Report		X			X					X	X	X	X	X
Self-Report Questionnaires §§		X					X					X		X
Usability and Satisfaction Survey					X							X*	X*	X*

* Subjects randomized to intervention arm only
** Three visits for witnessed doses; phase1: Week 1 and Week 2
§ Done per standard of care within 30 days of screening and captured whenever done during the course of the study
§§ Habitual self-control, self-efficacy beliefs, depression scale (PHQ-9), alcohol use (AUDIT) and drug use (DAST-10) questionnaires
λ Only subjects seen at UCSD AVRC will undergo collection of plasma for storage.

6.2 Evaluations

6.2.1 Pre-Entry Evaluations

Occur prior to the subject taking any study medications, treatments, or interventions.

6.2.1.1 Patient Registration

A coded patient identification number (PID) will be assigned to each patient screened for the study. PIDs should not be reassigned even if the patient fails to enter the study. The PID must be included on every case report form (CRF) and patient blood sample. BIT Core must maintain a master list of PIDs in a central location. The patient registration and inclusion/exclusion CRF must be completed on the online system.

6.2.1.2 Screening Clinical Labs

If plasma sputum, resistance testing, biochemistry, and hematology laboratories were obtained at a CLIA-approved lab no more than 90 days prior to screening and results are available then these labs do not need to be repeated. If these laboratories are not available from County records or medical records, then hematology, microbiology, chemistry, and pregnancy testing will be coordinated through the public health or AVRC laboratory and results will be tracked on the online database.

Screening entry evaluations in addition to screening laboratories will include documentation of TB, and medical and medication history with report of clinical assessment, including toxicity to TB treatment. Attitudes to mobile devices and internet usage, and ability to have a mobile device housed and charged at home will be ascertained. County healthcare workers will be asked about suitability of individual for inclusion in study. Once the screening laboratories and other evaluations have been done, the patient will be evaluated for study eligibility if all other entry criteria are satisfied.

If any laboratory values are outside the eligible range, the site may re-screen a patient on one occasion. However, the study will not pay for additional re-screening laboratories.

6.2.1.3 Enrollment

Once a subject is deemed as eligible at screening they will be consented for enrollment into the study.

6.2.1.4 Phase 1

The Day 0 evaluation should be scheduled within 45 days of the screening visit. Subjects will undergo training and instruction on the DHFS system and initiation of study drug with DOT. A clinical assessment including toxicity will take place on Day 0, Week 1, and Week 2. Subjects will be trained with the PDH system by study staff, including placing the patch under observation Day 0, Week 1, and Week 2. Subjects will begin DHFS IS-RM (or Rifamate with co-ingestion of an encapsulated edible sensor) on Day 0, which will be directly observed. Patients will have two study visits per week in Week 1 and Week 2 for directly observed ingestion of DHFS IS-RM (or Rifamate with co-ingestion of an encapsulated edible sensor). Monitoring for side effects will take place at visits. At the end of Week 2 the subject will have their plasma drawn and stored. All subjects will complete items on PDH system usability and satisfaction.

6.2.1.5 Randomization

Subjects who wish to continue and remain eligible will be randomized 2:1 to DHFS IS-RM or to the control group receiving standard of care (SOC). Randomization for treatment assignment will be done centrally via the electronic database.

If the subject is randomized to the control arm, public health will provide isoniazid and rifampin, or Rifamate and DOT as per their routine. Please remind the subject to fill medication and contact public health as soon as possible. Please contact public health to inform them subject has been randomized to SOC DOT. The public health nurse assigned to this case should be handed the cost analysis data collection materials. The public health nurse will collect and perform quality control of the DOT forms used, but the outreach workers that actually perform the DOT.

If the subject is randomized to the intervention arm they will be given a 14-30-day supply of treatment medication, DHFS IS-RM, from the AVRC pharmacy on the entry day.

6.2.2 On-Study Evaluations, Phase 2

6.2.2.1 Day 0

Subjects randomized to the intervention will undergo clinical assessment including toxicity. They will receive training and instruction will take place on home use of DHFS, continuous patch wearing, use of mobile device with application screen. They will be monitored for correct patch use. Pre-study interviews will collect subject attitudes to mobile devices

and internet usage, as well as their understanding of taking anti-mycobacterial medications thus far and what they would like to gain from use of the PDH monitoring system. Self-report questionnaires will be administered on a mobile device. The subject's plasma will be drawn and stored only if they are subjects seen at the AVRC. Cost data collected. The patient will be instructed to take IS-RM at the clinic. From this point on study personnel and public health workers will check adherence data provided by DHFS daily and perform adherence intervention as necessary.

6.2.2.2 Week 2 to Completion of Treatment/End of Study

All subjects will receive a clinical assessment with toxicity and side effect evaluation, completion of a self-report adherence questionnaire and cost data collection. In the intervention arm, adherence will be checked (by reviewing DHFS logs) by study staff and public health daily and intervention will be performed as appropriate. At Weeks 2, 4, 8, and every 4 weeks until treatment is completed/end of study, adherence questionnaires will be collected; for subjects seen at the AVRC, Phase 2 visits will include a blood draw with stored plasma. In the intervention arm, the DHFS system usability and satisfaction questionnaires will be collected at Week 8, Week 12, and every 4 weeks until treatment is completed/end of study. For all subjects, at Week 8 and at the end of study, self-report questionnaires will be completed on a mobile device.

6.2.3 Change in Anti-Mycobacterial Regimen

Intervention subjects who require a change their treatment regimen from DHFS IS-RM for any reason will be required to permanently discontinue their study regimens.

If the regimen change is due to microbiologic failure, the subject would be evaluated for microbiologic failure during physician clinic visits (Section 6.1).

6.2.4 Post-Treatment Evaluations

All randomized subjects will complete an end of study visit, which will occur when a subject has completed their TB treatment (up to a maximum of 12 months on study). If the sponsor ends the study early, the end of study visit evaluation will be completed, regardless of week number.

6.2.5 Premature Treatment Discontinuation

These evaluations are required at the subject's final visit if they stop the study any time before the end of their treatment.

6.2.6 Pregnancy

Women who become pregnant during the study will be required to permanently discontinue their study regimens and will not continue to be followed on study. They should be advised to seek best available medical care for their pregnancy according to U.S. TB treatment guidelines.

6.3 Special Instructions and Definitions of Evaluations

6.3.1 Documentation of TB

The research coordinator will obtain results of any TB cultures, smears, cytology, and histopathology, from County records and medical records, and record them in the CRF.

6.3.2 TB Drug Resistance Definition

Multi-drug-resistant tuberculosis (MDR-TB) is defined as tuberculosis that is resistant to at least isoniazid and rifampicin (RMP). Isolates that are multiply resistant to any other combination of anti-TB drugs but not to isoniazid and RMP are not classed as MDR-TB. Extensively drug-resistant tuberculosis is defined as TB that has developed resistance to at least rifampicin and isoniazid (resistance to these first line anti-TB drugs defines multi-drug-resistant tuberculosis, or MDR-TB), as well as to any member of the quinolone family and at least one of the following second-line anti-TB injectable drugs: kanamycin, capreomycin, or amikacin [22].

6.3.3 TB Drug Resistance Testing

MDR-TB is defined by clinical, microbiologic, or molecular testing [23].

6.3.4 Medical and Laboratory History

At screening, a medical history will be obtained and must be recorded in the source documents. The medical history should include detailed information on mycobacterial infection, clinical course, and drug susceptibilities.

6.3.5 Medication History

At screening, a medication history (**only if within last 30 days prior to entry**) with actual or estimated start and stop dates should be obtained and recorded in the source documents and the concomitant medication CRF, including:

- All prescription medications. Including medications taken for the treatment or prophylaxis of opportunistic infections.
- Non-prescription medications.

- Alternative therapies and/or dietary supplements.
- Allergies to any medications and their formulations must be documented.

6.3.6 Concomitant Medications

During study visits (see Section 6.1 for specific dates) all concomitant medications taken since the last visit will be recorded in the source documentation and entered into the concomitant medication log CRF.

6.3.7 Study Treatment Modifications

All modifications to study drug(s) including initial doses, patient-initiated and/or protocol-mandated interruptions, modifications, and permanent discontinuation of anti-mycobacterials need be recorded on the CRFs at each visit.

6.3.8 Clinical Assessments

Targeted Physical Exam

A targeted physical examination will be based on any signs or symptoms previously identified that the subject has experienced within 30 days of entry or since the last visit. This examination will be performed at Day 0 and at study visits, as specified in the Schedule of Events, and in instances of microbiologic failure/toxicity or premature treatment discontinuation if they should occur.

Height and Weight

Height and weight should be measured at study entry. Weight will be measured at every study visit.

Signs and Symptoms

All signs, symptoms, deaths, and toxicities must be documented in the subject's record. At entry, record all signs/symptoms experienced within 30 days of entry on the CRFs. For all other visits and at the time of occurrence of microbiologic failure, record all Grade $\geq$ 2 signs and symptoms and deaths on the CRFs that have occurred since the last visit. Any signs or symptoms that lead to a change in DHFS treatment or the investigator thinks could possibly be due to DHFS treatment, regardless of Grade, must be recorded on the CRF. The source document must include date of onset and date of resolution, but the CRF will only record prevalence of a given adverse event since the previous study visit.

Refer to the Division of AIDS Table for Grading Adult Adverse Experiences.

Diagnoses

The following should be recorded on the CRFs: TB diagnosis and co-morbidities. Any other diagnosis that is, in the opinion of the site investigator, associated with study product or DHFS system, should be recorded on the adverse event CRF. The source document must include date of diagnosis and date of resolution.

Vital Signs

Temperature, pulse, and blood pressure will be collected at all visits and kept as a part of the source document.

6.3.9 Laboratory Evaluations

For all visits record all Grade ≥ 2 laboratory values on the CRFs throughout the course of the study. All values, regardless of toxicity, of specific laboratories will also be recorded on the laboratory CRF; including: white blood cell count, neutrophil count, hemoglobin, platelets, blood urea nitrogen, creatinine, glucose, AST/ALT, alkaline phosphatase, total bilirubin.

Any laboratory toxicities that lead to a change in TB or DHFS treatment, regardless of Grade, must be recorded on the adverse event CRF.

Refer to the Division of AIDS Table for Grading Adult Adverse Experiences, which can be found on the ACTG website: https://actgnetwork.org/

Hematology

Laboratory values will be done per SOC at public health and recorded in the CRF.

Liver & Kidney Function Tests

Total bilirubin, AST (SGOT), ALT (SGPT), and alkaline phosphatase, BUN, creatinine and electrolytes (sodium, potassium, chloride, and bicarbonate) will be collated from the public health laboratory.

Pregnancy Test

For women with reproductive potential: Urine β-HCG (urine test must have a sensitivity of ≤ 50 mIU/mL) should be done at the site's local laboratory during screening, at study entry and whenever clinically suspected.

6.4 Off-Drug Requirements

Additional safety monitoring and reporting of serious adverse events (SAEs) thought to be at least possibly related to DHFS will be required upon completion or discontinuation

of study protocol regardless of whether a protocol follow-up visit is scheduled to occur. Follow-up will continue until the adverse event is resolved or stabilized.

7.0 CLINICAL MANAGEMENT ISSUES

7.1 Adverse Events

Adverse events will be captured by study personnel throughout a patient's participation in both the active portion of the protocol as well as the follow-up period. Wearable sensor-related adverse events will be captured from the onset of exposure (e.g. first patch application) to the end of device exposure (e.g. removal of last patch, or resolution of last wearable sensor-related AE, whichever is later). Ingestible sensor-related adverse events will be captured from the onset of exposure (e.g. first ingestion) to the end of device exposure (e.g. last ingestion, or resolution of last ingestible-sensor related adverse event).

Table 2. Adverse Event-Related Definitions

**Adverse Event (AE)**	Any undesirable medical event occurring in research subject, whether or not the event is considered related to the investigational device.
**Serious Adverse Event (SAE)**	An AE that results in death, is life-threatening (even if temporary in nature), results in a permanent impairment of body function or permanent damage to body structure, hospitalization, prolongation of hospitalization, or medical or surgical intervention to preclude permanent impairment of a body function or permanent damage to a body structure, or represent a serious medical event. An SAE may or may not be considered related to the investigational device.
**Anticipated Adverse Event (AAE)**	An AE that is listed in the clinical protocol or other study-related document that has been identified as a *potential* AE related to the investigational device or procedure being studied.
**Unanticipated Adverse Device Effect (UADE)**	Any serious adverse effect on health or safety or any life-threatening problem or death caused by, or associated with, a device, if that effect, problem or death was not previously identified in nature, severity or degree of incidence in the investigational plan or application or any other unanticipated serious problem associated with a device that relates to the rights, safety or welfare of subjects.

In some adverse event reports, the cause of the adverse event may not be immediately apparent, due to the overlapping nature of the anticipated adverse events from the DHFS and from the concomitant medications with which the patients will enroll in the study. In these cases, it is the investigator's responsibility to appropriately adjudicate the cause of the adverse events, taking into consideration the location, nature, onset, and duration of the adverse event, as well as the

known safety profiles of both the study device, study drug, and other concomitant drugs. The following table outlines the reporting algorithm to be used for Adverse Events.

Table 3. Adverse Event Reporting Algorithm, Investigator's (or Designee's) Responsibilities

**Type**	**Action**	**Communication**
**Non-Serious Adverse Event**	▪ Record on the CRFs	Written on CRF
**Serious Adverse Event (SAE), unrelated** to the device	▪ Record on the CRFs	Written on CRF
**Serious Adverse Event (SAE),** possibly or definitively related to the device, anticipated	▪ Record on the CRFs<br>▪ Preliminary report **to Proteus Digital Health within 3 days** (reporting days) of the investigator/site's becoming aware<br>▪ Full report submitted as additional information becomes available.<br>▪ Full report submitted **to the IRB within 10 working days** of the investigator/site's becoming aware	Verbal and written
**Unanticipated Adverse Device Effect (UADE)**	▪ Record on the CRFs<br>▪ Preliminary report **to Proteus Digital Health within 3 days** of the investigator/site's becoming aware<br>▪ Full report **to Proteus Digital Health** as additional information becomes available. Report **to the IRB as soon as possible, but within 10 working days** of the investigator/site's becoming aware	Verbal and written

Investigators should make the required reports to the manufacturer's Safety Officer:

Greg Moon, MD
Director of Clinical Affairs
Proteus Digital Health, Inc.
2600 Bridge Parkway, Suite 101, Redwood City, California 94065
Phone: (Office) 650-637-6111, (Mobile) 415-939-6425, Fax: 650-632-4071
Email: gmoon@proteusdh.com

Once the manufacturer has been notified of an adverse event, it will fulfill its reporting requirements to regulatory authorities per Proteus Digital Health SOP000794, *Adverse Events: Reporting Requirements for Investigational and Approved Devices*.

7.2 Toxicity Management

Adverse events due to Tuberculosis or the medical treatment of TB (Rifamate) will be handled per SOC and County criteria. Laboratories to monitor toxicity will be done per County criteria and will be recorded by study forms.

Toxicity Events Related to DHFS

No adverse effects have been observed after administering the PDH ingestible sensors to rats in a dose 1,000 times the maximum human daily dose (see Investigator's Brochure). Human testing to date has included more than 250 subjects, including healthy human subjects and subjects with TB and other chronic diseases, with more than 14,000 PDH ingestible sensors ingestions. More recent studies have occurred with no exclusion for pre-existing gastrointestinal diseases. Ingestions have occurred twice daily for as long as 12 weeks, and as many as 30 PDH ingestible sensors have been ingested in a single day with no adverse gastrointestinal effect. A single occurrence of nausea and vomiting (3.5% of all subjects) has been reported after multiple earlier prototype PDH ingestible sensors ingestions in a single day. Self-limited, minor, and localized skin irritation has occurred in 17.7% wearing the adhesive backed monitor. Adhesives with improved skin tolerability are being incorporated into newer versions of the PDH system. There have been no unanticipated adverse events and no serious adverse events related, or possibly related, to the PDH System.

Skin-related issues can occur with any adhesive medical product, including the common adhesive bandage, and the wearable sensor is no exception. To date, some subjects have experienced cutaneous AEs, in the form of transient, mild, macular, papular, or maculopapular erythematous rash at the wearable sensor site. These rashes appear to be consistent with irritant (i.e., non-immune mediated) contact dermatitis, a well-characterized and common phenomenon. (It is the same phenomenon that underlies the colloquially named "dishpan hands"). Each rash in prior system studies either resolved or was resolving significantly by the time of the final safety follow-up. Experience has shown that risk of rash can be significantly mitigated by rotating wearable sensor sites with no areas of overlap.

The sponsor is committed to the rigorous capture of skin AEs related to use of the system. However, not all issues are clinically significant. Thus, the following skin-related issues shall *not* be captured as AEs by study investigators:

1. Pruritis, *if not* associated with: a) visible skin changes, including excoriation or b) interruption of system use > 1 day.

2. Skin changes that would be routinely encountered through the wear of a common adhesive bandage (e.g., transient wrinkling/whitening of the skin under a bandage, mild hyperemia).

Verification that a Skin Issue has Occurred

Before being classified as an AE, skin issues must be verified. Verification can consist of either: a) direct visualization by the investigator or his/her designee or b) a photograph taken of the affected area. The most convenient means to accomplish the latter will be use of the mobile device issued to each study subject; site staff should train each subject regarding the mobile device camera feature as he/she enters a given study. The only exception is pruritis that leads to interruption of system use > 1 day, as there likely will be nothing to visualize.

Documenting Skin AE Evolution and Chronicity

Skin AEs frequently last longer than 1 day, so a means to document the course of the AE is described below. Skin findings that occur in the same location should be treated as a single AE, unless complete resolution at that location has been visualized and documented by the investigator. Moreover, a skin AE may evolve over its course (e.g., erythema progressing to papules, followed by scaling). The investigator should therefore name the skin AE based upon findings first encountered (e.g., "macular rash"). For each assessment episode that occurs in follow-up, record the following on the *Adverse Events Form* case report form:

1. Date of assessment

2. Actions taken

3. Outcome, using the Table TBD

4. Any new features of the same skin AE that emerge on its course to resolution (e.g., scaling, non-erythematous skin discoloration) captured within the "additional skin findings" data field for a given skin AE

5. Date of onset and date of resolution

Table 4. Procedure for Documenting Device-Related Skin AE Outcome

**Outcome**	**Definition**	**Action**	**CRF Used**
**Resolved**	No remaining signs of skin changes	Document date of resolution	Adverse Event form used (for interim visits) *OR* Follow-up Visit form (for final study visit)
**Resolving**	Skin changes have improved but are still discernible	Document as "resolving"<br>List any new skin findings<br>Must continue surveillance through last study visit	Adverse Event form used (for interim visits) *OR* Follow-up Visit form (for final study visit)

Outcome	Definition	Action	CRF Used
**Unresolved**	Skin changes have not improved or have worsened	Document as "unresolved"<br>List any new skin findings<br>Must continue surveillance through last study visit	Adverse Event form used (for interim visits) *OR* Follow-up Visit form (for final study visit)
**Resolved with Sequelae**	Original skin issues and all subsequent skin features that were present throughout course have resolved, but a condition as a consequence of the skin AE emerged	Document as "resolved with sequelae"<br>List the sequelae	Adverse Event form used (for interim visits) *OR* Follow-up Visit form (for final study visit)

On the CRF used for the last study visit (usually the safety follow-up visit), all skin AEs—except those that have not previously been documented as "resolved"—shall have a *final categorization* of outcome ("resolved," "resolving," "unresolved," or "resolved with sequelae").

Non-Erythematous Skin Discoloration

This finding has been noted infrequently in studies of the system. Following an initially erythematous rash, the skin can take on a brownish hue in the areas of prior erythema. This should not be considered a new skin AE, as it is a continuation of the previous AE. If detected, the investigator should list "non-erythematous skin discoloration" as a new skin finding in the associated data field. On each subsequent assessment, the investigator must make the determination of "resolved," "resolving," "unresolved," or "resolved with sequelae."

8.0 CRITERIA FOR DISCONTINUATION

8.1 Criteria for Treatment Discontinuation

- Drug-related toxicity (see Section 7.2, Toxicity Management).
- Requirement for prohibited concomitant medications (see Section 5.5.2)

8.2 Criteria for Discontinuation from the Study

- Pregnancy or breast-feeding.
- Active drug or alcohol use or dependence that, in the opinion of the investigator, would interfere with adherence to study requirements.
- Failure by the subject to attend three consecutive clinic visits during the follow-up portion may result in discontinuation.
- Request by the subject to withdraw.

- Request of the primary care provider if s/he thinks the study is no longer in the best interest of the subject.
- Clinical reasons believed life threatening by the physician, even if not addressed in the toxicity management of the protocol.
- Subject judged by the investigator to be at significant risk of failing to comply with the provisions of the protocol as to cause harm to self or seriously interfere with the validity of the study results.
- At the discretion of the AVRC, investigator, or sponsors.

9.0 STATISTICAL CONSIDERATIONS

9.1 General Design Issues

This study is a prospective, two phase, open-label feasibility study with lead in (Phase 1) and randomized (Phase 2) components to evaluate the utility of the DHFS for monitoring TB treatment. In the first phase, all subjects will receive DHFS IS-RM (or Rifamate with co-ingestion of an encapsulated edible sensor) and a series of doses will be witnessed in order to determine the detection accuracy of the system as well as to evaluate subject acceptance and ability to use the system. In the randomized phase, those randomized to intervention will continue to use the DHFS IS-RM and control subjects will receive SOC DOT.

9.2 Endpoints

9.2.1 Primary Endpoints

Phase 1 (1.2.1) Positive Detection Accuracy (PDA)

PDA is defined as DHFS detection of the ingestion of a dose of IS-RM (or Rifamate with co-ingestion of an encapsulated edible sensor) when compared to a witnessed ingestion of the same dose of medication. For purposes of PDA analysis, a dose is defined as a single IS-RM capsule. PDA will be based upon dose ingestions that occur during concurrent use of DHFS and DOT during the planned Phase 1 study visits. A minimum of 280 dose ingestions are required to generate sensitivity and specificity of the System.

Phase 2 (1.2.2) Percentage of Witnessed Doses

This will be calculated as the total number of witnessed doses ingestions divided by the total number of prescribed witnessed dose ingestions. For the DHFS, witnessed doses by DHFS is defined as the total number of dose ingestions confirmed by DHFS, divided by the total number of prescribed witnessed dose ingestions for TB treatment. For SOC, witnessed doses will be defined as the total number of dose ingestions that are actually witnessed by a healthcare worker, divided by the total number of prescribed witnessed dose ingestions for TB treatment. This endpoint will be calculated from Phase 2, Day 0 to Weeks 4, 8 and end of study.

9.2.2 Secondary Endpoints

(1.2.3) The definition of 80% adherence will be completion of ≥ 80% of witnessed doses.

(1.2.4) Lapse in prescribed witnessed doses will be defined by missing 1 or more prescribed witnessed doses; the duration of lapses will be defined by the number of sequential missed prescribed witnessed doses in a row.

(1.2.5) The total cost of treatment for DHFS IS-RM and SOC DOT will be defined based on specific data collected for that purpose.

(1.2.6) Adverse events of the DHFS system or DOT will be defined as any grade 1 or higher DHFS or DOT related adverse clinical and laboratory events.

(1.2.7) Responses to individual questions and grouped question responses (i.e., summary metrics across questions) will be defined to evaluate subject satisfaction with the DHFS system.

(1.2.8) Responses to individual questions and grouped question responses (i.e., summary metrics across questions) will be defined to evaluate healthcare worker and physician satisfaction with DHFS and DOT.

(1.2.9) Individual question and summary metrics will be defined for each of the self-reported instruments: 1) habitual self-control, 2) self-efficacy beliefs, 3) PHQ-9, 4) AUDIT, and 5) DAST-10. If standardized scores exist (e.g., for the PHQ-9), these scoring systems will be used to define the endpoint.

(1.2.10) The definition of 80% adherence will be completion of > 80% of witnessed doses.

9.3 Randomization

Subjects will be randomized 2:1 to DHFS or SOC. Randomization will be done centrally via the study database.

9.4 Sample Size and Accrual

Phase 1: For phase 1, both groups will use the DHFS for 2 weeks, and we will estimate the confidence interval around an estimate of the detection accuracy of the DHFS by taking the ratio of the sensor-enabled ingestion recorded events compared to the witnessed doses. Assuming that each of 75 subjects completes 2 weeks of DHFS dosing and has a total of three witnessed doses each week (a total of 450 dosing events) and that the overall rate of positive detection is 95%, then the 95% confidence interval around the point estimate of 95% accurate detection would be +/- 2.0%. If the detection accuracy was 97%, then the 95% confidence interval around the point estimate of 97% accurate detection would be +/- 1.6%.

Phase 2: The primary aim of phase 2 is to assess the percentage of prescribed witnessed doses completed over the entire study period for each subject and to compare the DHFS arm to the DOT arm. This metric captures the persistent efficacy of the DHFS ingestion sensor system and allows comparison to SOC DOT of varying intervals (5 times, 3 times, or 2 times per week that may occur in the SOC). Sample size calculations were based on a two-sided, two-sample t-test to compare the differences in adherence percentages by treatment arm using different mean/standard deviation scenarios. Calculations were performed using the R statistical package (version 2.14.0) (www.r-project.org).

In choosing a sample size for this pilot study, we considered issues of cost, feasibility, as well as how large an effect size would be of interest to spur further study of this intervention in this population. Assuming a two-sided alpha of 0.05, an adherence percentage in the DOT arm of 90%, and a common standard deviation of 6%, we will need 75 participants (50 DHFS arm and 25 DOT arm) to detect a 5% difference (i.e. 95% adherence percentage in DHFS arm) with 92% power.

9.5 Monitoring

The study team will review all adverse events during the study by cumulative reports, on a monthly basis. Adverse events will be graded using the ACTG toxicity grading scale and recorded using standard AE electronic data capture. The study investigators will monitor safety events in aggregate on monthly team calls. An independent Data Safety and Monitoring Board will not be used for this study.

9.6 Analyses

This section briefly describes the planned statistical analysis. The Statistical Analysis Plan (SAP), which will be finalized prior to database lock, provides further details. In case the language in this section differs from the language in the SAP, the SAP takes precedence.

In general, analyses will incorporate the modified intent-to-treat principle, namely, all randomized participants dispensed at least one dose of medication will be included in the analysis. No adjustments for multiple comparisons will be made for secondary analyses, and a p-value of 0.05 will be considered statistically significant.

Demographic data, concomitant medications, site of TB infection, adverse events prior to entry into study due to TB medications and past medical history will be collated and described for all study subjects and presented in aggregate for phase 1 and by randomized group for phase 2.

9.6.1 Analysis for Primary Outcomes

Phase 1 (Detection Accuracy of the DHFS)

The primary analysis of the positive detection accuracy will be done by calculating the number of correct sensor detected IS-RM (or Rifamate with co-ingestion of an encapsulated edible sensor) ingestions divided by the total number of actual witnessed ingestion events with a 95% confidence interval. The data will be reviewed to detect false positive events (recorded ingestion when none should have occurred), especially on days where an observed dose is given and ingestion was recorded at another time. False positive and false negative ingestions will be determined as well as true positive ingestions. An intent-to-treat (all inclusive analysis) will be done that includes all events but will exclude doses that were either known to be not ingested or ingested during the period of time when the patch was known not to be worn. An additional adjusted analysis will use the same definition, but will exclude doses that were taken during a time period when the patch was not functioning properly, either by malfunction or user error.

Phase 2 (Percentage of Witnessed Doses)

A mixed effects regression model will be used to determine if the WOT intervention arm will produce greater percentage of witnessed doses compared to the DOT standard of care arm at treatment end. This model will include time, treatment arm, and time-by-treatment arm interaction terms. In addition, any covariates, which are simultaneously unbalanced at baseline and associated with the outcome, will be included in the model. Time will be treated as a categorical variable. A significant treatment effect will be concluded if the p-value for the time-by-treatment interaction contrast in the model is $<=0.05$. Permutation tests may be implemented to verify that valid p-values will be obtained even if model assumptions are not correct. Note that the linear mixed model seamlessly accommodates different times of measurement as well as missed measurements and data from subjects who are lost to follow-up. Generalized estimating equation (GEE) models will be used as the sensitivity analysis method.

Note that when considering percentages of witnessed doses over the entire study period in aggregate, this model reduces to a multiple linear regression, and the independent variable of interest is the treatment arm.

9.6.2 Analysis for Secondary Outcomes

All secondary measures will be summarized descriptively by study arm and overall. Mixed effects regression models analogous to the primary analysis methodology will be applied for the secondary outcomes. There will be no adjustments made for multiple testing corrections for secondary outcomes. P-values less than 0.05 will be considered statistically significant.

The percentage of subjects completing > 80% prescribed witnessed dosing for TB treatment using DHFS and SOC, respectively will be calculated and compared using two sample t-tests (or non-parametric equivalent) for the duration of follow-up. The time to first evidence of < 80% adherence will be calculated in the DHFS only as this may not be available in the SOC data.

The number of lapses in witness doses will be compared between DHFS and SOC using Wilcoxon rank sum test. The longest duration of lapse in witnessed treatment will be calculated and compared between the DHFS and SOC groups.

Data collected and model considerations for the costs analyses will include: 1) number of visits from outreach worker divided by length of time on study, 2) outreach worker hours spent per subject divided by length time on study; 3) distanced traveled per subject divided by length of time on study; 4) distance traveled by outreach worker divided by length of time on study, and 6) number of hospitalizations divided by length of time on study. Patient travel costs and lost wages will be reported. Wage costs with benefits of County workers and time spent per case will be calculated. Sensitivity analyses as appropriate with variation of costs and rates of failure derived from pre-existing County data will be performed of DHFS and DOT and will include all costs related to TB treatment, laboratory monitoring, assessment and treatment of adverse events, costs of DHFS and equipment associated with DHFS.

Adverse events related to study procedures (either DHFS or SOC DOT) will be graded using standard ACTG grading tables and listed by organ system and severity. Of note, GI symptoms, hepatic enzyme abnormalities, or other expected toxicities of Rifamate or TB disease itself that were pre-existing and were not exacerbated during the study will be recorded, but will not be adjudicated as related or possibly related to DHFS.

Subject responses regarding satisfaction with the DHFS and DOT will be reported on post study questionnaires regarding their experience with the DHFS and the usability of the system, using summary statistics. Questionnaire responses by subjects will be examined by study reviewers from AVRC and will present a set of recommendations regarding the usability of the system. Areas evaluated may include ease of use, time needed to use the system, perceived personal benefit, negative impressions, risk of disclosure of health status and changes to quality of life. Analyses of individual questions as well as summary metrics across questions will be explored. Analyses will use descriptive statistics. If there is variation in satisfaction with the DHFS, the demographic characteristics potentially related to different levels of satisfaction will be explored.

Healthcare and physician satisfaction with the DHFS will be reported quantitatively, using non-standard customized questionnaires to collect information regarding their experience with the DHFS and the usability of the system, using summary statistics. Questionnaire responses by providers will be examined by study reviewers from AVRC and make a set of recommendations

regarding the usability of the system. DOT satisfaction will be reported using questionnaires.

Summary statistics will be used to define the results of self-reported instruments. Comparisons between groups will use Fisher's exact test or Wilcoxon rank sum test as appropriate.

Reported clinical and demographic characteristics, as well as responses to self-reported questionnaires, will be compared for subjects with < 80% adherence to those with greater than 80% adherence. Comparisons will include: demographics, disease characteristics, and self- reported metrics. Self-reported metrics will be defined based on the individual instrument. For example, depression will be defined as a PHQ-9 score > 5.

10.0 PHARMACOLOGY PLAN INCLUDING INTENSIVE PK SUBSTUDY

The PK substudy described in this Section was completed in November 2014, and will not enroll any additional participants. See below Section 10.4 Preliminary Results for early data from this substudy.

The focus of the pharmacology substudy of AHF TB 001 is to prospectively evaluate the PK parameters, determined by sequential, within subject intensive PK sampling, of isoniazid (INH) and rifampin concentrations derived from dosing with Rifamate when given in native format compared to when dosed with the over-encapsulated PDH IS system.

10.1 Pharmacology Substudy Objectives

10.1.1 To determine if pharmacokinetic parameters (CL/F, AUC, C_{min}, and C_{max}) for INH and rifampin are similar within subjects when dosed with over-encapsulated ingestion sensor-enabled Rifamate (IS-RM) compared to dosing with non-encapsulated Rifamate.

10.1.2 To explore whether the distributions of these parameters are comparable to published (i.e., historical) data.

10.1.3 To explore the associations between these parameters and measurements of adherence as measured by the IS-RM.

10.2 Pharmacology Substudy Design

A subset of 12 patients on rifampin and isoniazid will be enrolled in this two-period, randomized intensive pharmacokinetic (PK) substudy. They will meet all the inclusion/exclusion criteria detailed in this protocol. Prior to entry into the PK substudy, subjects will be required to be on five times per week DOT with 300mg isoniazid and 600mg rifampin for a minimum of one week. Upon entry, subjects will be randomized to one of two different sequences of initiating over-encapsulated versus native Rifamate. Thus, subjects will complete one of the following: 1) Complete 14 days of treatment with the study medication (over-encapsulated Rifamate with IS),

undergo pharmacokinetic sampling, then go onto 2 weeks of native Rifamate, followed by a second PK sampling. 2) Complete 14 days of treatment with native Rifamate, undergo pharmacokinetic sampling, then go onto two weeks of over-encapsulated Rifamate with IS followed by a second PK sampling. The schema below describes the two PK sampling sequences and timeframe:

Table 5. PK Substudy Sampling Sequences

	Randomize	Period 1: study day 0 to 14	PK sampling Day 14	Period 2: day 15 to day 28	PK sampling Day 28
PK sub-study; N = 12 subjects		Rifamate over-encapsulated IS	24 hr intensive PK sampling	Rifamate 2 capsules daily	24 hr PK sampling
		Rifamate 2 capsules daily	24 hr PK sampling	Rifamate over-encapsulated IS	24 hr intensive PK sampling

If necessary, subjects may separate Period 1 and Period 2 of the PK substudy at the discretion of the Study PI.

Subjects who have previously completed Phase 1 alone may elect to participate in the PK substudy after individual case review and at the discretion of the Study PI. In this case the subject would repeat Phase 1.

10.2.1 Recruitment

Public health nurses will inform their eligible patients of the PK substudy and refer interested patients to the study coordinators for more information and/or screening. The study coordinator will ensure that patients understand that participation in the PK substudy is voluntary and that PK substudy visits are in addition to the main study clinic visits. Recruitment for this sub-study will only take place in San Diego County.

10.2.2 Informed Consent

Eligible subjects choosing to participate in the PK substudy will sign a separate consent form for the substudy. The PK substudy informed consent form is in addition to the main protocol consent form. Subjects must first sign the main study consent form before signing the PK substudy consent form.

10.2.3 Sampling

In this substudy, subjects will either initiate Phase 1 of the main study, taking over-encapsulated Rifamate with the ingestible sensor (IS), or initiate native

Rifamate for 14 days. On Day 14, when the subject has taken at least 14 uninterrupted doses of over-encapsulated Rifamate with IS, they will have an intensive PK sampling with blood draws occurring over a 24-hour period. The date and time of the previous three doses of Rifamate will be recorded on the CRF. An IV lock will be placed for blood draws. A pre-dose C_{trough} blood sample will be obtained and the daily dose of over-encapsulated Rifamate with IS, or native Rifamate will be administered with 8 ounces of water. The subject should remain fasting for 2 hours and then can eat as desired. PK samples will be collected at eight time points: 0 (prior to dosing), 1, 2, 3, 4, 6, 8 and 24 hrs. Two 6ml heparin tubes will be drawn per time point. Also, we will have 1 ml of discard per time point to clear the IV lock. Therefore, about 100 ml of blood will be drawn per participant per study day (24 hrs).

Table 6. PK Substudy Schedule of Events

		Plasma sampling for INH + RIF								
Visit	Switch to native Rifamate ***OR*** over-encapsulated Rifamate with IS	0 hour (pre-dose)	Witnessed dose of Rifamate	1	2	3	4	6	8	24
Day 14		X	X	X	X	X	X	X	X	X
Day 15	**X**									
Day 28		X	X	X	X	X	X	X	X	X

After the first intensive PK sampling, subjects will be placed on native Rifamate or over-encapsulated Rifamate with IS for two weeks. After at least 14 days on native Rifamate, or over-encapsulated Rifamate with IS, the PK substudy patients will again have blood samples drawn for an intensive PK analysis. Once again, an IV lock will be placed, and blood samples taken at 0 (prior to dosing), 1, 2, 3, 4, 6, 8 and 24 hrs. The fasting and medication dosing directions will be the same as above.

Plasma will be collected and processed from all subjects at the time points noted above. Samples will be stored at -70°C (or colder) until shipped frozen on dry ice to the laboratory, where they will be maintained at -70°C (or colder) until analysis. All samples will be stored. They may be processed individually or in batches. Only subjects with two weeks of uninterrupted, over-encapsulated Rifamate dosing witnessed by the DHFS and DOT will be sampled. Any subject who has missed any doses of study treatment within the 3 days prior to a scheduled PK sampling visit should have the PK sampling visit rescheduled within 1 week.

Samples will be analyzed for isoniazid and rifampin drug concentration levels

either individually or in batches at a central specialized laboratory. Substudy subjects who demonstrate bioequivalence for isoniazid and rifampin levels between the native Rifamate and over-encapsulated Rifamate with IS will be allowed to continue onto Phase 2, if they remain eligible, at the discretion of the Study PI.

10.2.4 Required Data

At each sampling visit, the subject's weight, and time of administration of the last three doses of each of the Rifamate doses will be collected. Height will be recorded for all subjects at the first PK visit. Information about the last intake of food/drink must also be recorded for all subjects.

10.3 Primary and Secondary Data, Modeling, and Data Analysis

All samples will be identified by a unique patient ID and study number only at the specialized testing laboratory. The plasma concentrations of isoniazid and rifampin in the plasma of subjects from the study will be measured using validated assays (such as HPLC or UV) as appropriate. De-identified data will be compiled and analyzed using non-compartmental and, as appropriate, a compartmental pharmacokinetic model. Individual estimates of primary and secondary parameters (t1/2, CL/F, C_{max}, C_{trough} and AUC) will be determined.

The individual PK parameters will be compared within subjects by calculating the geometric mean ratio of isoniazid and rifampin PK parameters from the native to the over-encapsulated dosing intervals. The 90 percent confidence interval will be calculated around these ratios using appropriate models, including those that include factors for gender, weight, and BMI.

10.4 Preliminary Results

Patients were enrolled in a substudy of AHF TB 001 to evaluate the PK parameters for both native isoniazid and rifampin (combined together in Rifamate) compared to over-encapsulated IS-RM. Twelve patients were enrolled in the PK study and had blood drawn for sampling after completing 14 days of both native Rifamate and IS-RM. Results show that levels of both isoniazid and rifampin in IS-RM compared to isoniazid and rifampin levels in native Rifamate met the criteria for bioequivalence. Given these findings of bioequivalence, the PK substudy is complete and no longer enrolling patients.

11.0 DATA COLLECTION AND MONITORING AND ADVERSE EXPERIENCE REPORTING

11.1 Records to Be Kept

Electronic Case report forms (eCRF) will be provided via a secure, user-authenticated study website hosted by the UCSD Center for AIDS Research Bioinformatics and Information Technologies (BIT) Core. These eCRFs will be used by study staff with proper permissions to enter patient data into the study database as needed. Subjects will not be identified by name on any CRFs, but rather by a confidential PID provided by the BIT Core upon study screening.

The following describes the capacity and function of the UCSD BIT Core for all study-related data security and management:

The BIT Core is comprised of three faculty members, five professional programmers, and several other support staff with world-class expertise in infectious disease bioinformatics, computational biology, software development, website design and hosting, and clinical study data management. In addition to this experienced staff, the BIT Core also possesses extensive computational resources including a +700 processor Linux Beowulf MPI cluster, seven rack-mounted web servers, and two fault-tolerant encrypted 16TB RAID6 data storage arrays. This hardware is hosted in a secure computing environment co-located at the UCSD San Diego Supercomputing Center (SDSC) and AVRC. The collective resources of the BIT Core will be made available to this study under the direction of Dr. Jason A. Young, the BIT Core biomedical data management scientific lead.

11.1.1 Central Database System

All data management for this study will be provided by the Open Source Clinical Content Management System (OCCAMS) developed by the BIT Core. OCCAMS provides integrated, flexible, and secure tools for complex clinical study data management using only a web browser. This includes: 1) an intuitive drag-and-drop eCRF editor, 2) methods for automatically displaying these eCRFs according to user-defined study protocol schedules, 3) easy-to-use interfaces for direct online data entry with integrated QA workflows and accrual monitoring, 4) a powerful database backend with data and form auditing capabilities, 5) an integrated specimen collection and tracking system with optional use of barcode labeling, and 6) flexible data import and export modules compatible with popular statistical packages such as R, SAS, etc. Furthermore, OCCAMS is built atop Plone, the most mature (first launched in 2001), secure (best record of any major CMS), compatible (runs on every major operating system), and actively developed (+340 core developers worldwide) open source content management system available today (plone.org). As such, OCCAMS utilizes built-in Plone functionalities including secure login and robust user management with granular permissions that are under continual security scrutiny by the open source community as a whole.

11.1.2 Clinical Data Receipt, Storage, Quality Control, Retrieval

Study personnel will use OCCAMS from a web browser to manage all data and specimen collection, storage, quality control, and retrieval. Each staff member will be provided a login and password and will be assigned to the appropriate role-based groups using the UCSD campus active directory. Membership in these role-based groups will provide users with the necessary web views to complete their assigned tasks on a need to know basis. For example, nurse permissions will only allow for viewing and entering of data into eCRFs, QC permissions will only allow for editing and verification of data entry, and laboratory permissions will only allow the collection and management of specimens. However, these roles

and views are extremely flexible, are not mutually exclusive, and can be easily managed from within OCCAMS by study supervisors with appropriate permissions, to meet specific study needs.

11.1.3 Receipt

Data and specimens will be directly entered into the database by clinical staff via OCCAMS eCRF and specimen collection workflows. Electronic CRFs will be designed using the occams.form module, whereas specimen collection and aliquot procedures will be defined in the occams.lab module. Following review, the eCRFs and specimen types will be assigned for collection to study visits as outlined in the Schedule of Events (see Section 6.1). Upon study initiation, OCCAMS will then automatically queue clinical staff to enter/collect the appropriate data and specimen(s) for each patient visit.

11.1.4 Storage

All data will be stored in an Entity-Attribute-Value database similar to standards used in commercial clinical data systems, but implemented using the open source database software postgreSQL and SQLAlchemy. Designed specifically for OCCAMS, this database (occams.datastore) allows for the precise tracking of all changes to eCRFs and data over time, providing exhaustive auditing functionality. The database is also split into two components: a protected health information (PHI) component for storage of personally identifiable data as defined by the Privacy Rule of the HIPAA Section 164.154, and a non-PHI component for all other data storage needs. This separation enhances data security by limiting access to sensitive parts of the database whenever possible. Furthermore, all databases are hosted from fault-tolerant encrypted RAID6 data storage arrays, with secure nightly synchronization between co-located sites.

11.1.5 Quality Control

Built-in OCCAMS quality control workflows and views will be used to ensure data accuracy throughout the study. Specifically, OCCAMS provides eCRF status descriptions that indicate where in the quality control workflow a particular eCRF resides. The “Pending-Entry” status denotes a patient visit has occurred and the clinical staff has yet to enter data for the eCRF required at that visit. Upon entry of this data, the eCRF is automatically moved to a “Pending-Review” status where it then becomes accessible to quality assurance staff for verification. Once verified for accuracy by quality assurance staff, the eCRF then enters a “Reviewed” status indicating it is ready to be included in data analysis. The flow of eCRFs through these statuses can be monitored in real-time from various reporting views accessible to staff with the appropriate permissions.

11.1.6 Retrieval

OCCAMS provides methods for both viewing and downloading data collected though eCRFs on a form-by-form basis. Files can be downloaded in csv format, which can then be easily imported into standard statistical packages (R, SAS, etc.) for offline analysis. By default, data viewing and download permissions are restricted to investigator and analyst roles, but can be expanded to others as needed. Regarding specimen retrieval, the occams.lab module provides query functionality allowing specimen search by type, date, and PID, as well as a specimen inventory system that supports the tracking of specimens using computer-generated barcoded labels.

11.1.7 Data Security Plans

The BIT Core ensures data security though a robust security model consisting of a multi-level hardware/software firewalled network, secure hardware and software encryption, data auditing and access tracking procedures, and completely redundant services hosted from two physically separated server facilities. All primary production machines are located behind a hardware firewall at the SDSC on the UCSD main campus in a server facility with biometric-based restricted access, temperature and humidity monitoring, seismic protection, 24/7 generator power backup, and continuous CCTV camera surveillance. For data and service redundancy in case of hardware or power failure at SDSC, secondary backup machines are located behind a hardware firewall at the AVRC on the UCSD medical campus in a server room with key card-based restricted access, temperature and humidity monitoring, and 24/7 CCTV camera surveillance. All communication between server facilities occurs over a gigabit UCSD line, encrypted using secure shell (SSH) on a Virtual Private Network (VPN) with strict access control. All web servers hosted by the BIT Core are virtualized and backed up routinely so they can be reinstated quickly on new hardware if needed. The BIT Core systems administrator and other key personnel are automatically notified by e-mail and SMS of any server abnormalities.

11.1.8 HIPAA/FISMA Compliance

Our approach to research information security is designed to comply with the latest requirements of HIPAA and FISMA regulations. For FISMA compliance, this includes documentation of management, operational, and technical processes used to secure the physical and virtual infrastructure based on the National Institute of Standards and Technology (NIST) Special Publication 800-53 and compliance with the Revision 3 standards for the Security Categories and Levels of “Moderate” as outlined by FIPS 199. To date, we have instituted: 1) a detailed categorization of information and information systems comprising our network, 2) a network map detailing all systems and services, 3) standardization of server images using CentOS-6 with SELinux enabled, 4) clear documentation of file permissions, 5) centralized user authentication using Active Directory, 6) encryption of all user desktop OSX file systems and data backup, 7) nightly

encrypted backup of all data to secure co-located sites at the SDSC and AVRC, 8) extensive logging of user access, and 9) a sandboxed environment for all software development.

11.1.9 Data Labeling, Storage, Handling, and Disposal

Specimens collected throughout the proposed contract will be managed using the specimen management component of OCCAMS, called occams.lab. This component provides integrated workflows to clinical staff for facilitating the collection of specimen(s) per study visit, splitting of these specimens into user-defined numbers of aliquot, generation of barcoded labels for aliquot and specimen tracking, and specimen check-out workflows for shipment and disposal as required. For example, occams.lab has already been successfully used to manage over 200,000 specimens collected as part of HIV studies at the AVRC (including PBMCs, plasma, serum, urine, anal swabs, and CSF). For this study, lab assistants and phlebotomists will be responsible for reviewing and organizing the collection, processing, barcode labeling, transporting, and cryostoring of all samples. Lab assistants will be responsible for data entry and maintenance of the specimen inventories using occams.lab specimen query tools and auditing tools.

11.2 Project Management by BIT Core

11.2.1 Data Management and Tracking

Clinical site monitoring will be done by AVRC staff. Well-developed data monitoring operating procedures include monitored elements and timelines, procedures for generation and resolution of data queries, and reporting, including quality assurance workflows built into OCCAMS and available to appropriate study staff via the web. Review of regulatory documents and individual patient records and consent forms will occur bi-annually. Queries from the eCRF will be resolved and compared to source documentation. Clinical monitoring for all patients will include review of regulatory files, consent forms, inclusion criteria, and major endpoint verification. Monthly reports showing patients screened, early terminations, and late visits are shared with the protocol team, as well as listings of adverse events and assessment of data quality reports.

11.2.2 Previous Experience and Timeliness

The BIT Core has extensive experience in biomedical data management for infectious disease-related clinical trials. Dr. Young, the BIT Core scientific and strategy lead for biomedical informatics projects, has hands-on experience managing data needs for complex, multi-site clinical studies. Under the supervision of Dr. Young, other BIT Core team members that will be actively involved in this contract will include Mr. Marco Martinez (senior programmer) and Ms. Fang Wan (data manager), both of whom have relevant private sector and academic work experience. OCCAMS currently handles all data management needs for three ongoing clinical research studies at the AVRC, consisting of more

than 10,000 patient screens, 1,000 patient enrollments, 200,000 specimens, and 5GB clinical data originating as far back as 1996. Collectively, these projects currently support more than 50 clinical staff working at four sites throughout Southern California (UCSD, USC, UCLA, and Long Beach Department of Health and Human Services). Furthermore, OCCAMS is continually being expanded and refined to meet new clinical research needs as they arise, having been designed from the beginning to be modular and extensible.

11.3 Role of Data Management

Instructions concerning the recording of study data on eCRFs will be provided by the BIT Core. It is the responsibility of the BIT Core to assure the quality of computerized data for this study.

11.4 Clinical Site Monitoring and Record Availability

Monitors will review the individual participant records, including consent forms, eCRFs, supporting data, laboratory specimen records, and medical records (physician progress notes, nurse notes, hospital charts), to ensure protection of study subjects, compliance with the protocol, and accuracy and completeness of records. The monitors also will inspect regulatory files to ensure that regulatory requirements are being followed, and the site pharmacy to review product storage and management.

The investigator will make study documents (e.g., consent forms, drug distribution forms, CRFs) and pertinent hospital or clinic records readily available for inspection by the local Institutional Review Board (IRB), the site monitors, the Food and Drug Administration (FDA), the pharmaceutical sponsor(s), or the sponsor's designee for confirmation of the study data.

11.5 Serious Adverse Experience Reporting

Serious adverse experiences must be documented on the Serious Adverse Event (SAE) Reporting Form and submitted to the BIT Core.

12.0 HUMAN SUBJECTS

12.1 Institutional Review Board Review and Informed Consent

This protocol and the informed consent document and any subsequent modifications will be reviewed and approved by the Institutional Review Board (IRB) or ethics committee responsible for oversight of the study. A signed consent form will be obtained from the subject (or parent, legal guardian, or person with power of attorney for subjects who cannot consent for themselves, such as those below the legal age of consent). The consent form will describe the purpose of the study, the procedures to be followed, and the risks and benefits of participation. A copy of the consent form will be given to the subject, parent, or legal guardian, and this fact will be documented in the subject's record.

12.2 Subject Confidentiality

All laboratory specimens, evaluation forms, reports, and other records that leave the site will be identified by coded number only to maintain subject confidentiality. All records will be kept locked. All computer entry and networking programs will be done with coded numbers only. Clinical information will not be released without written permission of the subject, except as necessary for monitoring by the IRB, AVRC, BIT Core, or Proteus Biomedical.

12.3 Study Discontinuation

The study may be discontinued at any time by the BIT Core, IRB, and the industry supporter(s).

13.0 PUBLICATION OF RESEARCH FINDINGS

Publication of the results of this trial will be governed by AVRC policies. Any presentation, abstract, or manuscript will be made available for review by Proteus Biomedical prior to submission.

14.0 BIOHAZARD CONTAINMENT

As the transmission of HIV, TB and other blood-borne pathogens can occur through contact with contaminated needles, blood, and blood products, appropriate blood and secretion precautions will be employed by all personnel in the drawing of blood and shipping and handling of all specimens for this study, as currently recommended by the Centers for Disease Control and Prevention and the National Institutes of Health.

All dangerous goods materials, including diagnostic specimens and infectious substances, must be transported using packaging mandated by CFR 42 Part 72. Please refer to instructions detailed in the International Air Transport Association Dangerous Goods Regulations.

15.0 REFERENCES

1. Weis SE, Foresman B, Matty KJ, Brown A, Blais FX, Burgess G, King B, Cook PE, Slocum PC. Treatment costs of directly observed therapy and traditional therapy for Mycobacterium tuberculosis: a comparative analysis. *Int J Tuberc Lung Dis* 1999; 3:976-984.

2. Vernon AA, Iademarco MF. In the treatment of tuberculosis, you get what you pay for. *Am J Respir Crit Care Med* 2004; 170:1040-1042.

3. Chang KC, Leung CC, Yew WW, Chan SL, Tam CM. Dosing schedules of 6-month regimens and relapse for pulmonary tuberculosis. *Am J Respir Crit Care Med* 2006; 174:1153-1158.

4. Bova CA, Fennie KP, Knafl GJ, Dieckhaus KD, Watrous E, Williams AB. Use of electronic monitoring devices to measure antiretroviral adherence: practical considerations. *AIDS Behav* 2005; 9:103-110.

5. Wendel CS, Mohler MJ, Kroesen K, Ampel NM, Gifford AL, Coons SJ. Barriers to use of electronic adherence monitoring in an HIV clinic. *Ann Pharmacother* 2001; 35:1010-1015.

6. Olivieri NF, Matsui D, Hermann C, Koren G. Compliance assessed by the Medication Event Monitoring System. *Arch Dis Child* 1991; 66:1399-1402. PMCID: PMC1793394

7. Laine C, Newschaffer CJ, Zhang D, Cosler L, Hauck WW, Turner BJ. Adherence to antiretroviral therapy by pregnant women infected with human immunodeficiency virus: a pharmacy claims-based analysis. *Obstet Gynecol* 2000; 95:167-173.

8. Young-Xu Y, Chan KA. Pooling overdispersed binomial data to estimate event rate. *BMC Med Res Methodol* 2008; 8:58. PMCID: PMC2538541

9. Wu JR, Moser DK, Chung ML, Lennie TA. Objectively measured, but not self-reported, medication adherence independently predicts event-free survival in patients with heart failure. *J Card Fail* 2008; 14:203-210. PMCID: PMC2751635

10. Stroupe KT, Teal EY, Tu W, Weiner M, Murray MD. Association of refill adherence and health care use among adults with hypertension in an urban health care system. *Pharmacotherapy* 2006; 26:779-789.

11. Feinstein AR. On white-coat effects and the electronic monitoring of compliance. *Arch Intern Med* 1990; 150:1377-1378.

12. Feldman SR, Camacho FT, Krejci-Manwaring J, Carroll CL, Balkrishnan R. Adherence to topical therapy increases around the time of office visits. *J Am Acad Dermatol* 2007; 57:81-83.

13. Alcoba M, Cuevas MJ, Perez-Simon MR, Mostaza JL, Ortega L, Ortiz de Urbina J, Carro JA, Raya C, Abad M, Martin V. Assessment of adherence to triple antiretroviral treatment including indinavir: role of the determination of plasma levels of indinavir. *J Acquir Immune Defic Syndr* 2003; 33:253-258.

14. Vrijens B, Vincze G, Kristanto P, Urquhart J, Burnier M. Adherence to prescribed antihypertensive drug treatments: longitudinal study of electronically compiled dosing histories. *BMJ* 2008; 336:1114-1117. PMCID: PMC2386633

15. Wolf MS, Davis TC, Osborn CY, Skripkauskas S, Bennett CL, Makoul G. Literacy, self-efficacy, and HIV medication adherence. *Patient Educ Couns* 2007; 65:253-260.

16. Gross R, Tierney C, Andrade A, Lalama C, Rosenkranz S, Eshleman SH, Flanigan T, Santana J, Salomon N, Reisler R, Wiggins I, Hogg E, Flexner C, Mildvan D, Team ACTGAS. Modified directly observed antiretroviral therapy compared with self-administered therapy in treatment-naive HIV-1-infected patients: a randomized trial. *Arch Intern Med* 2009; 169:1224-1232. PMCID: PMC2771688

17. DiCarlo L, Moon G, Intondi A, Duck R, Frank J, Hafazi H, Behzadi Y, Robertson T, Costello B, Savage G, Zdeblick M. A digital health solution for using and managing medications: wirelessly observed therapy. *IEEE Pulse* 2012; 3:23-26.

18. Au-Yeung KY, Robertson T, Hafezi H, Moon G, DiCarlo L, Zdeblick M, Savage G. A networked system for self-management of drug therapy and wellness. Available in: *Proceedings of Wireless Health 2010.* doi 10.1145/1921081.1921083

19. Au-Yeung KY, Moon GD, Robertson TL, Dicarlo LA, Epstein MS, Weis SE, Reves RR, Engel G. Early clinical experience with networked system for promoting patient self-management. *Am J Manag Care* 2011; 17:e277-287.

20. Belknap R, Weis S, Brookens A, Au-Yeung KY, Moon G, DiCarlo L, Reves R. Feasibility of an ingestible sensor-based system for monitoring adherence to tuberculosis therapy. *PloS One* 2013; 8:e53373. PMCID: PMC3538759

21. Blumberg HM, Burman WJ, Chaisson RE, Daley CL, Etkind SC, Friedman LN, Fujiwara P, Grzemska M, Hopewell PC, Iseman MD, Jasmer RM, Koppaka V, Menzies RI, O'Brien RJ, Reves RR, Reichman LB, Simone PM, Starke JR, Vernon AA. American Thoracic Society/Centers for Disease Control and Prevention/Infectious Diseases Society of America: Treatment of tuberculosis. *Am J Respir Crit Care Med* 2003; 167:603-662.

22. World Health Organization. WHO global task force outlines measures to combat XDR-TB worldwide. 2006. Available at www.who.int/mediacentre/news/notes/2006/np29.

23. Centers for Disease Control and Prevention Division of Tuberculosis Elimination. Report of expert consultations on rapid molecular testing to detect drug-resistant tuberculosis in the United States. Last updated September 1, 2012. Available at www.cdc.gov/tb/topic/laboratory/rapidmoleculartesting.
